# Integrative bioinformatic and experimental analysis reveals prognostic and immunological roles of MEX3 family genes in glioma

**DOI:** 10.3389/fimmu.2025.1654036

**Published:** 2026-01-14

**Authors:** Xuezhong Zhang, Hui Shang, Tingting Chu, Kaihui Sha

**Affiliations:** 1 Department of Laboratory Medicine, Zibo Central Hospital, Zibo, Shandong, China; 2 Shandong Jincheng Pharmaceutical Group Co., Ltd., Zibo, Shandong, China; 3 Department of Rehabilitation Medicine, Dongying People’s Hospital (Dongying Hospital of Shandong Provincial Hospital Group), Dongying, Shandong, China; 4 School of Nursing, Binzhou Medical University, Binzhou, Shandong, China

**Keywords:** bioinformatics analysis, enrichment analysis, experimental analysis, glioma, immune infiltration, MEX3 family genes

## Abstract

**Background:**

Glioma is a highly heterogeneous and aggressive malignancy of the central nervous system, and reliable molecular biomarkers are urgently needed to improve prognostic stratification and guide therapeutic decision-making. The MEX3 family of RNA-binding proteins has been implicated in tumorigenesis and post-transcriptional regulation; however, their comprehensive roles in glioma remain poorly understood.

**Methods:**

Integrated bioinformatic analyses were performed using transcriptomic and clinical data from TCGA, CGGA, and GEO cohorts to evaluate the expression profiles, diagnostic and prognostic value, genetic alterations, molecular interactions, immune infiltration characteristics, and functional pathways associated with MEX3A, MEX3B, MEX3C, and MEX3D. Protein-protein interaction networks, gene set enrichment, and co-expression analyses were conducted to explore potential biological mechanisms. A MEX3-related prognostic risk model was constructed and validated in independent datasets. Drug sensitivity correlations were analyzed using public pharmacogenomic resources. In addition, *in vitro* experiments, including qRT-PCR, western blotting, proliferation, migration, and invasion assays, were performed in U251 and LN229 glioma cell lines to functionally validate the bioinformatic findings.

**Results:**

All four MEX3 family members were significantly upregulated in glioma tissues compared with normal controls and demonstrated strong diagnostic performance. Distinct prognostic patterns were observed, with MEX3D consistently identified as an independent predictor of poor overall, disease-specific, and progression-free survival. MEX3 genes were associated with diverse genetic alterations and were enriched in pathways related to RNA processing, cell cycle regulation, and cancer-associated signaling. Immune analyses revealed significant correlations between MEX3 expression and multiple immune cell populations as well as immune checkpoint molecules, suggesting potential roles in shaping the glioma immune microenvironment. A MEX3-related co-expression-based prognostic model showed robust survival-predictive ability and remained effective in external validation cohorts. Functional assays confirmed that silencing individual MEX3 genes significantly inhibited glioma cell proliferation, migration, and invasion *in vitro*.

**Conclusions:**

This study provides a comprehensive characterization of the MEX3 family in glioma, demonstrating their dysregulation, prognostic relevance, immune associations, and functional contributions to malignant phenotypes. Among them, MEX3D emerges as a particularly promising prognostic biomarker. These findings establish a foundation for future mechanistic and translational studies exploring MEX3 family members as potential biomarkers or therapeutic targets in glioma.

## 1 Introduction

Gliomas are the most common primary malignant tumors of the central nervous system (CNS) and comprise a heterogeneous group of neoplasms originating from glial cells, including astrocytoma, oligodendroglioma, ependymoma, anaplastic astrocytoma, and glioblastoma (GBM) (1, 2). Low-grade gliomas (LGG) generally display slower clinical progression and prolonged survival compared with high-grade lesions (3). Conversely, GBM accounts for more than half of all glioma cases and is associated with a median survival of less than 15 months despite aggressive multimodal treatment (4). This clinical variability reflects the substantial biological heterogeneity of gliomas and highlights the need for additional molecular markers to improve tumor classification and prognostic assessment. Consistent with this need, the 2021 WHO classification of CNS tumors incorporated molecular features as integral components of diagnosis (5).

Recent investigations have identified a growing number of genes involved in glioma initiation and progression, underscoring the importance of discovering biomarkers with improved diagnostic and prognostic performance (2). The Muscle EXcess 3 (MEX3) gene family-comprising MEX3A, MEX3B, MEX3C, and MEX3D-encodes evolutionarily conserved RNA-binding proteins characterized by two K-homology domains and a RING-type E3 ubiquitin ligase domain (6). These proteins are pivotal in regulating mRNA stability, localization, and translation, influencing key biological processes such as embryonic development, immune system modulation, and tumor formation (6–8). Dysregulated MEX3 expression has been documented in breast (9), lung (7), gastric (10), and hepatocellular cancers (11), where individual family members may exert oncogenic or tumor-suppressive functions depending on the biological context.

Although the roles of MEX3 proteins in glioma remain insufficiently defined, emerging mechanistic evidence suggests potential involvement in immune-associated signaling and RNA quality control pathways that may be relevant to CNS tumor biology. In non-CNS malignancies, MEX3A has been implicated in modulating JAK-STAT and NF-κB signaling, in regulating nonsense-mediated decay of tumor-suppressive transcripts, and in interacting with RNA sensors such as RIG-I (12). Limited studies in glioma have reported upregulation of MEX3A and associations with enhanced proliferative and invasive capacities, suggesting that MEX3 family members may influence both intrinsic RNA-metabolic programs and interactions with the tumor microenvironment (13). These preliminary observations support further investigation of the MEX3 family in CNS tumors. RNA-binding proteins (RBPs) broadly regulate post-transcriptional gene expression, and several RBPs have been implicated in glioma pathogenesis. HNRNPK modulates transcriptional and post-transcriptional pathways that promote tumor cell proliferation and invasion (14), whereas FXR1 is upregulated in high-grade gliomas and contributes to the stabilization of oncogenic RNAs (15). These examples illustrate the potential relevance of RBP-mediated regulation in glioma biology and provide rationale for examining additional RBP families.

Advances in high-throughput sequencing and computational analysis have facilitated the integration of multi-omic datasets, enabling systematic characterization of tumor-associated genes (16–20). In the present study, we conducted a comprehensive bioinformatics analysis of MEX3A, MEX3B, MEX3C, and MEX3D in glioma, and evaluated their expression patterns, prognostic significance, genomic alterations, and potential functional pathways across publicly available datasets. This study aims to clarify the roles of MEX3 family members in glioma and to assess their potential utility as diagnostic and prognostic biomarkers.

## 2 Methods

### 2.1 Reclassification under WHO CNS5

To improve the accuracy and clinical relevance of our analysis, we reclassified glioma samples based on the 2021 World Health Organization Classification of Tumors of the Central Nervous System (WHO CNS5), which emphasizes integrated molecular diagnostics. First, clinical and molecular data for glioma samples were obtained from the UCSC XENA TCGA-LGG and TCGA-GBM datasets. Due to the UCSC XENA platform not yet reflecting WHO CNS5 updates, we complemented the datasets with additional molecular information-specifically, IDH mutation status, 1p/19q codeletion status, and MGMT promoter methylation status-retrieved from cBioPortal and TCGA Pan-Cancer Atlas annotations. We then reassigned each glioma sample into the following categories according to WHO CNS5 criteria: Astrocytoma, IDH-mutant: samples with IDH mutation and no 1p/19q codeletion. Oligodendroglioma, IDH-mutant and 1p/19q-codeleted: samples with both IDH mutation and 1p/19q codeletion. Glioblastoma, IDH-wildtype: samples with wild-type IDH and histological or molecular features consistent with glioblastoma, including TERT promoter mutation, EGFR amplification, or +7/−10 chromosomal changes.Samples lacking key molecular markers necessary for classification were excluded from subgroup analyses to maintain data integrity.

### 2.2 Expression profile analysis, survival analysis, and clinicopathological features of MEX3 family genes in glioma

To investigate the expression patterns of MEX3 family genes in glioma, we utilized an integrated dataset from the UCSC XENA platform (https://xenabrowser.net) (21). This included uniformly processed, TPM−formatted RNA−seq data from TCGA glioma samples and corresponding normal brain tissue samples from the GTEx project. All expression values were log_2_−transformed prior to statistical analysis to stabilize variance. Glioma samples from TCGA were reclassified according to the WHO CNS5 (2021) standards. Visualization of MEX3 family genes expression from UCSC XENA database was performed using the “ggplot2” package in R. Furthermore, the relationship between MEX3 family genes mRNA expression and clinicopathological features of glioma was assessed through R’s ggplot2 package. Kaplan-Meier survival analysis was implemented to evaluate overall survival (OS), disease-specific survival (DSS), and progression-free interval (PFI) based on MEX3 family genes expression levels (22). This was conducted using the “Survival” package in R software, with hazard ratios (HR) and p-values determined and reported. To assess the performance of binary classification models, receiver operating characteristic (ROC) curves were analyzed. The ROC curve measures classification effectiveness by comparing sensitivity and specificity, while the area under the curve (AUC) quantifies the model’s accuracy, with values closer to 1 indicating superior classification performance. The “pROC” package was employed for ROC curve analysis (23).

### 2.3 cBioPortal analysis of MEX3 family genes in glioma

Genetic alterations in MEX3 family genes within the TCGA dataset were examined via cBioPortal (24). The analysis included all MEX3 family members (MEX3A, MEX3B, MEX3C and MEX3D) from the TCGA pan-cancer atlas cohort for LGG and GBM. The names of the MEX3 family genes were entered into the designated search field, allowing retrieval of mutation data from both “OncoPrint” and “Cancer Types Summary” models within the cBioPortal platform.

### 2.4 Protein-protein Interaction network construction of MEX3 family genes in glioma

The GeneMANIA platform (http://genemania.org) represents a powerful bioinformatics resource for investigating functional associations and molecular interactions among gene products. This comprehensive database integrates interaction data encompassing 166,691 genes and 660 million documented relationships across nine model organisms. In our study, we utilized GeneMANIA to predict potential interaction partners of MEX3 family proteins in Homo sapiens and constructed a protein-protein interaction network to explore their biological relationships. Spearman correlation analysis was performed to investigate the associations between MEX3 family genes and predicted potential interaction genes in glioma, with results visualized using the “ggplot2” and “pheatmap” packages.

### 2.5 Immune-related analysis of MEX3 family genes in glioma

The ssGSEA algorithm was applied to evaluate immune cell infiltration, covering 24 immune cell types in glioma tissue samples (25). Correlation analyses between MEX3 family genes expression levels, immune cells, and immune checkpoint markers (PDCD1, CTLA4, and PD-L1) were performed using Spearman correlation analysis. These associations were visually represented with the “ggplot2” and “pheatmap” packages. To compare immune cell enrichment in glioma samples with high and low MEX3 family genes expression, the Wilcoxon rank-sum test was conducted.

### 2.6 Gene set enrichment analysis

Gene set enrichment analysis (GSEA) was performed using R software (version 3.6.3) to identify pathways potentially associated with the expression of MEX3 family genes in glioma. The cohort was divided into high and low expression groups based on the median expression level, with MEX3 family genes expression serving as the phenotype indicator. Each GSEA run included 1,000 permutations. The “c2.cp.v7.2.symbols.gmt” gene set from the Molecular Signatures Database was used as the reference. Pathways were considered significantly enriched if they met the following criteria: false discovery rate (FDR)< 0.25, P-value< 0.05, and normalized enrichment score (NES) > 1. Pathways with the highest NES values were prioritized for further interpretation.

### 2.7 Functional and pathway analysis

Glioma patient gene expression data were sourced from the TCGA database. Pearson correlation coefficients (P< 0.01, |R| > 0.4) identified genes co-expressed with MEX3 family members. Venn diagram analysis was used to visualize the overlapping relationships among MEX3A, MEX3B, MEX3C and MEX3D. To explore MEX3 family genes-related biological pathways and functions, the “clusterProfiler” package facilitated Kyoto Encyclopedia of Genes and Genomes (KEGG) (26) and Gene Ontology (GO) analyses (27) of co-expressed genes. GO analysis was categorized into biological process (BP), molecular function (MF), and cellular component (CC).

### 2.8 Identification of prognostic genes and Prognostic model construction based on MEX3 family co-expression genes

To systematically assess the prognostic value of MEX3 family co-expression network genes in glioma, we performed univariate Cox proportional hazards regression analysis (significance threshold: P< 0.05) using the “survival” R package. Potential multicollinearity among predictors was addressed through LASSO-penalized Cox regression analysis, implemented via the “glmnet” (v4.1.7) R package, to optimize model parsimony. A nomogram for individualized survival probability prediction was constructed using the “rms” R package.

Risk scores were computed using centralized and standardized mRNA expression data from the glioma training cohort. The score was derived as follows: Risk score= $\sum_{i}^{n}xiyi$. Where X represents the LASSO-derived coefficient for each pyroptosis-related gene, and Y denotes the expression level of MEX3 family co-expressed genes. Patients were stratified into high- and low-risk groups based on the median risk score. Overall survival (OS) differences between groups were evaluated using Kaplan-Meier analysis. Model predictive performance was quantified by time-dependent ROC curves generated with the “timeROC” package. Covariates included gender, age, race, WHO grade, IDH status, 1p/19q codeletion, Primary therapy outcome, histological type, and lasso.risk.score.

### 2.10 Drug sensitivity assessment

To evaluate drug sensitivity, we retrieved the IC50 values of 481 small-molecule compounds from the CTRP database, along with the corresponding mRNA expression data of the MEX3 family genes. Pearson correlation analysis was performed to assess the association between mRNA expression levels and drug sensitivity. The resulting P-values were adjusted for false discovery rate (FDR). To identify chemicals associated with the MEX3 gene family (MEX3A, MEX3B, MEX3C, MEX3D), we queried the Comparative Toxicogenomics Database (CTD; https://ctdbase.org/). Each gene (MEX3A, MEX3B, MEX3C, and MEX3D) was individually entered into the search bar, followed by clicking “Search” and selecting the “Chemicals” category. This retrieved all compounds linked to each MEX3 family member. The resulting datasets were downloaded, and overlapping interactions were visualized using a Venn diagram. The three-dimensional (3D) structures of the relevant compounds were obtained from PubChem (https://pubchem.ncbi.nlm.nih.gov/).

### 2.11 Validation of MEX3 family genes expression using the GEO and CGGA databases

To further validate the expression patterns and prognostic models of MEX3 family genes in glioma, independent transcriptomic datasets from the CGGA (mRNAseq_693 and mRNAseq_325) (http://www.cgga.org.cn/) (28), and GEO (GSE16011) databases were analyzed to verify their expression profiles (29).

### 2.12 *In vitro* cell culture and protocos

The human glioma cell line U251 and LN299, obtained from the American Type Culture Collection (ATCC, Manassas, VA, USA), was cultured under standard conditions. Cells were maintained in Dulbecco’s Modified Eagle Medium (DMEM) supplemented with 10% fetal bovine serum (FBS) and 1% penicillin-streptomycin, and incubated at 37°C in a 5% CO_2_-humidified atmosphere. Cells at the logarithmic growth phase were used in subsequent assays. For transfection experiments, cells were seeded into 6-well plates at a density of 3 × 10^5^ cells per well, incubated for 24 hours, and then transfected using Lipofectamine 2000 reagent (Invitrogen, New York, CA) according to the manufacturer’s instructions. Transfection reagents included either a scrambled RNA sequence as a negative control (NC) or independent, gene-specific small interfering RNAs (siRNAs) targeting each gene individually: MEX3A (si-MEX3A), MEX3B (si-MEX3B), MEX3C (si-MEX3C), and MEX3D (si-MEX3D). After transfection, cells were cultured for an additional 24 hours before being subjected to downstream analyses. The siRNA sequences were designed using Thermo Fisher’s online primer design tool and synthesized by Shanghai GenePharm (Shanghai, China). Detailed sequence information is provided in **Supplementary Table 1**.

### 2.13 Quantitative real-time polymerase chain reaction

Quantitative PCR was carried out in accordance with standard protocols. SYBR Green PCR Master Mix (Mei5 Biotechnology Co., Ltd) was used for amplification on the QuantStudio 5 system (Applied Biosystems, Carlsbad, CA). The thermal cycling conditions were as follows: initial denaturation at 95°C for 30 seconds, followed by 40 amplification cycles of 95°C for 5 seconds and 60°C for 30 seconds. Relative mRNA levels of MEX3A, MEX3B, MEX3C, and MEX3D were normalized to 18S ribosomal RNA, and data analysis was performed **Supplementary Table 2**.

### 2.14 Western blotting

WB was performed to assess protein expression levels. At 72 h post-transfection, cells were collected and lysed in RIPA buffer (Epizyme, Shanghai, China) containing protease inhibitors (Sollerbauer, Beijing, China). Protein concentration was determined with a BCA Protein Assay Kit (Epizyme), and lysates were equalized to ensure consistent loading across samples. Proteins were resolved by SDS-PAGE and subsequently transferred onto PVDF membranes (Millipore, Billerica, USA). After blocking with 5% non-fat milk at room temperature for 2 h, membranes were probed overnight at 4°C with the following primary antibodies: anti-MEX3A (1:800, CBG122A, CUSABIO), anti-MEX3B (1:1000, SA240318X09, ORIGEN), anti-MEX3C (1:1000, 15I0053, affinity), anti-MEX3D (1:1000, BE01081275, BIOSS), and anti-β-actin (1:2000, 20536-1-AP, Proteintech). Signal detection was carried out using an ECL chemiluminescence detection system (Epizyme).

### 2.15 Cell proliferation assay

Cell proliferation was assessed using two complementary approaches: the Cell Counting Kit-8 (CCK-8; Dojindo, Kumamoto, Japan) and colony formation assays. For the CCK-8 assay, cells were seeded into 96-well plates at a density of 1 × 10³ cells per well and cultured for 0H, 24H, 48H, 72H, or 96H. At each time point, 10 µl of CCK-8 reagent was added to each well and incubated for 2H at 37°C, after which absorbance at 450 nm was recorded using a microplate reader.

For colony formation assays, 400 cells were plated in six-well plates and maintained for 2 days. Colonies were fixed with 4% paraformaldehyde for 20 min, stained with crystal violet for 15 min, rinsed with water, and air-dried before being counted under a light microscope. All assays were conducted in triplicate to ensure reproducibility.

### 2.16 Cell migration assay

Cell migratory ability was examined using wound healing and transwell assays. For the wound healing assay, cells were grown to approximately 90% confluence, and a linear scratch was made using a 200 µL pipette tip. After washing with PBS, cells were cultured in serum-free medium. Wound closure was photographed at 0H, 12H, and 24H using an inverted microscope at identical positions. Migration was quantified by measuring the reduction in wound width. For the transwell assay, 1×10^4^ cells suspended in medium with 5% FBS were added to the upper chamber of a 24-well transwell insert (8-µm pore size; Corning, USA). The lower chamber contained medium supplemented with 10% FBS as a chemoattractant. After 24H, cells remaining on the upper surface were removed with a cotton swab, while migrated cells on the lower surface were fixed, stained with 0.5% crystal violet, and imaged at 100× magnification. Cell counts were obtained from five randomly selected microscopic fields per well.

### 2.17 Statistical analysis

For normally distributed data, values were expressed as means ± standard deviation. Wilcoxon rank-sum and Wilcoxon signed-rank tests in SPSS 23.0 were utilized to compare MEX3 family genes expressions between normal and glioma tissues. The relationship between MEX3 family genes expressions and clinicopathological variables was analyzed using a chi-square test. Kaplan-Meier survival analysis with the log-rank test was conducted to assess patient survival. Both univariate and multivariate Cox proportional hazards regression analyses were employed to evaluate the prognostic significance of the variables. Statistical significance was defined as *P< 0.05, **P< 0.01, and ***P< 0.001.

## 3 Results

### 3.1 Expression and prognostic value

**Figure 1:**
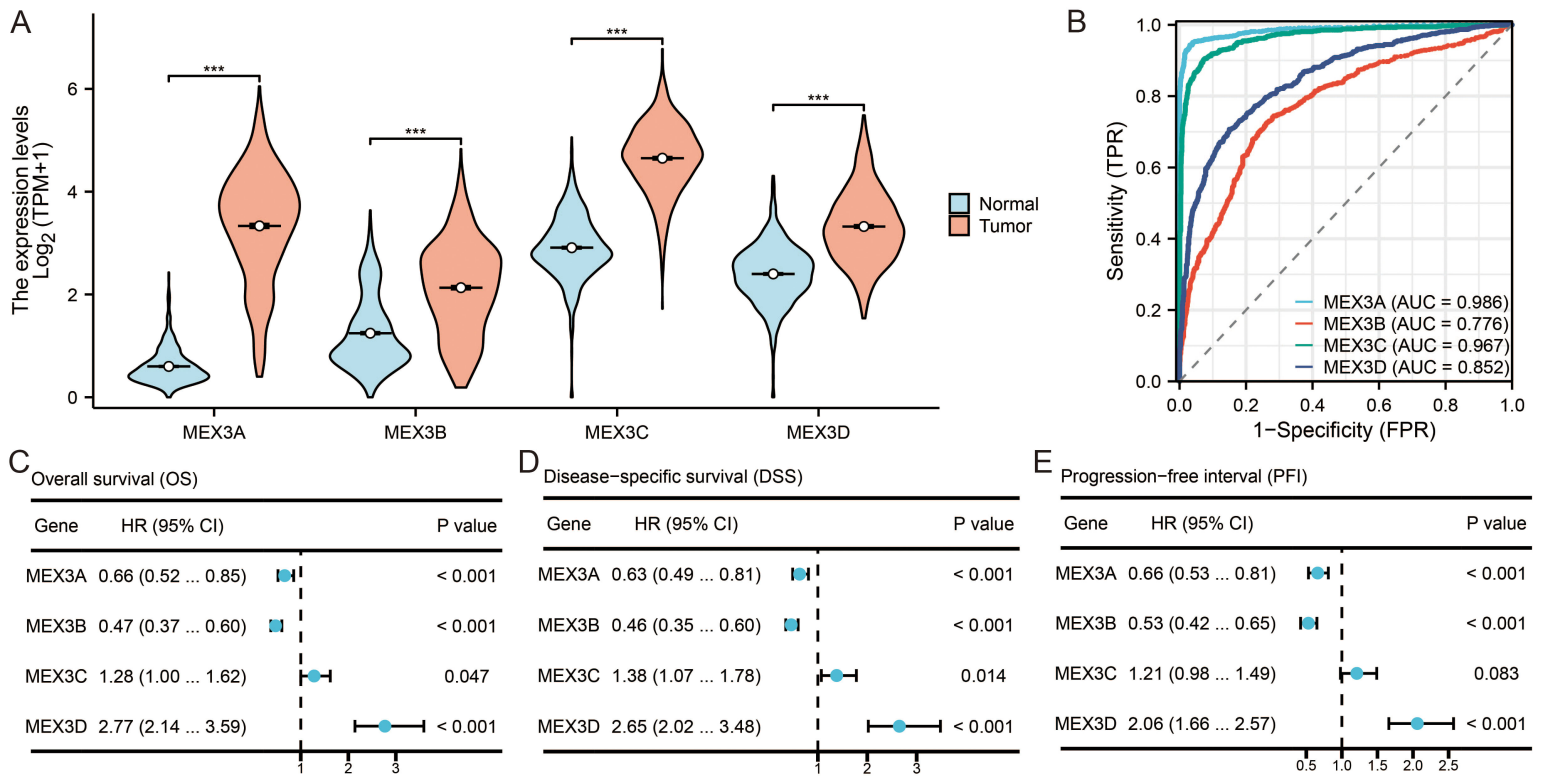
Expressions, receiver operator characteristic (ROC) curves and survival analysis of MEX3 family genes in glioma. **(A)** The expression of MEX3A, MEX3B, MEX3C and MEX3D in glioma tissue and normal tissue (***p < 0.001). **(B)** The (ROC) curves of MEX3 family members (MEX3A, MEX3B, MEX3C and MEX3D) of glioma. **(C–E)** Survival analysis (OS, DSS and PFI) of MEX3 family genes in glioma patients.

Analysis of UCSC XENA datasets revealed distinct expression profiles of the MEX3 gene family (MEX3A, MEX3B, MEX3C, and MEX3D) across 1157 normal and 689 glioma samples (sample sizes provided in **Supplementary Table 3**). All four genes were significantly upregulated in glioma tissues compared with normal controls (P< 0.001, **Figure 1A**). To assess their diagnostic utility, ROC curve analysis was performed (**Supplementary Table 4**). MEX3A (AUC = 0.986), MEX3C (AUC = 0.967), MEX3D (AUC = 0.852), and MEX3B (AUC = 0.776) demonstrated strong discriminatory performance between glioma and normal tissues (**Figure 1B**). The prognostic relevance of MEX3 family members was further evaluated using Kaplan-Meier survival analyses based on TCGA data (**Figures 1C–E**, **Supplementary Table 5**). Low MEX3A and MEX3B expression was consistently associated with shorter OS, DSS, and PFI (all P< 0.05). Conversely, high MEX3D expression predicted significantly poorer OS, DSS, and PFI (P< 0.05). Elevated MEX3C expression was similarly linked to reduced OS and DSS (P< 0.05); however, its association with PFI did not reach statistical significance (P = 0.083).

Across tumor grades, MEX3A showed its highest expression in Grade 3, with significantly higher levels than in Grades 2 and 4. MEX3B expression decreased markedly in Grade 4 compared with Grades 2 and 3. MEX3C expression was elevated in Grade 4 relative to Grade 2, while MEX3D demonstrated a progressive increase from Grade 2 to Grade 4 (**Supplementary Figure 1A**). Stratification by molecular features revealed distinct patterns. MEX3A and MEX3B were significantly upregulated in IDH-mutant (**Supplementary Figure 1B**) and 1p/19q-codeleted (**Supplementary Figure 1D**) glioma, whereas MEX3D was downregulated in IDH-mutant tumors and MEX3C/MEX3D showed reduced expression in 1p/19q-codeleted cases. Across histological subtypes, MEX3A and MEX3B were expressed at lower levels in glioblastoma than in astrocytoma and oligodendroglioma. MEX3C was reduced in oligodendroglioma compared to the other subtypes, while MEX3D showed highest expression in glioblastoma (**Supplementary Figure 1C**). Further analysis revealed distinct expression patterns of MEX3 genes across molecular subtypes (classical, mesenchymal, proneural). MEX3A was lower in mesenchymal vs. classical/proneural, and lower in proneural vs. classical. MEX3B was higher in proneural vs. classical/mesenchymal, with no difference between classical and mesenchymal. MEX3C was higher in classical vs. mesenchymal/proneural, with no difference between proneural and mesenchymal. MEX3D was lower in mesenchymal vs. classical/proneural, with no difference between classical and proneural (**Supplementary Figure 1E**). We further validated the upregulation of MEX3A−D in glioma using two independent datasets: CGGA (**Supplementary Figure 2A**) and GSE16011 (**Supplementary Figure 2B**), both confirming significantly elevated MEX3A−D expression in tumor tissues.

We analyzed the relationships between MEX3 family genes expressions and key clinicopathological variables in glioma (**Supplementary Table 6**). Gender and race showed no significant associations with MEX3A, MEX3B, or MEX3C, while MEX3D displayed only weak trends. Age correlated with MEX3A, MEX3B, and MEX3D expression, with higher levels of MEX3A/B in younger patients and elevated MEX3D in older patients. WHO grade, IDH mutation, and 1p/19q codeletion were strongly associated with expression patterns of MEX3A, MEX3B, and MEX3D, whereas MEX3C showed limited variation. MEX3A/B were enriched in lower-grade, IDH-mutant, and 1p/19q-codeleted tumors, while MEX3D was elevated in grade 4, IDH-wildtype, and non-codeleted tumors. MEX3C was downregulated in 1p/19q-codeleted gliomas but otherwise showed no significant differences. Across histological subtypes, MEX3A/B were expressed at higher levels in astrocytoma and oligodendroglioma, whereas MEX3D was most abundant in glioblastoma. MEX3C expression remained largely unchanged. Primary therapy outcome was not significantly associated with MEX3 gene expression.

To further identify prognostic risk factors in glioma patients, univariate and multivariate Cox regression analyses were performed (**Supplementary Tables 7**–**9**). Univariate Cox analysis showed that age, WHO grade, IDH status, 1p/19q codeletion, primary therapy outcome, and MEX3A-D expression were significantly associated with OS. Multivariate analysis identified age, WHO grade, IDH status, and primary therapy outcome as independent prognostic factors. Notably, elevated MEX3D expression remained independently associated with poorer OS and was further validated for DSS and PFI, indicating that MEX3D is an independent predictor of poor prognosis in glioma.

### 3.2 Mutation and protein-protein interaction network

**Figure 2:**
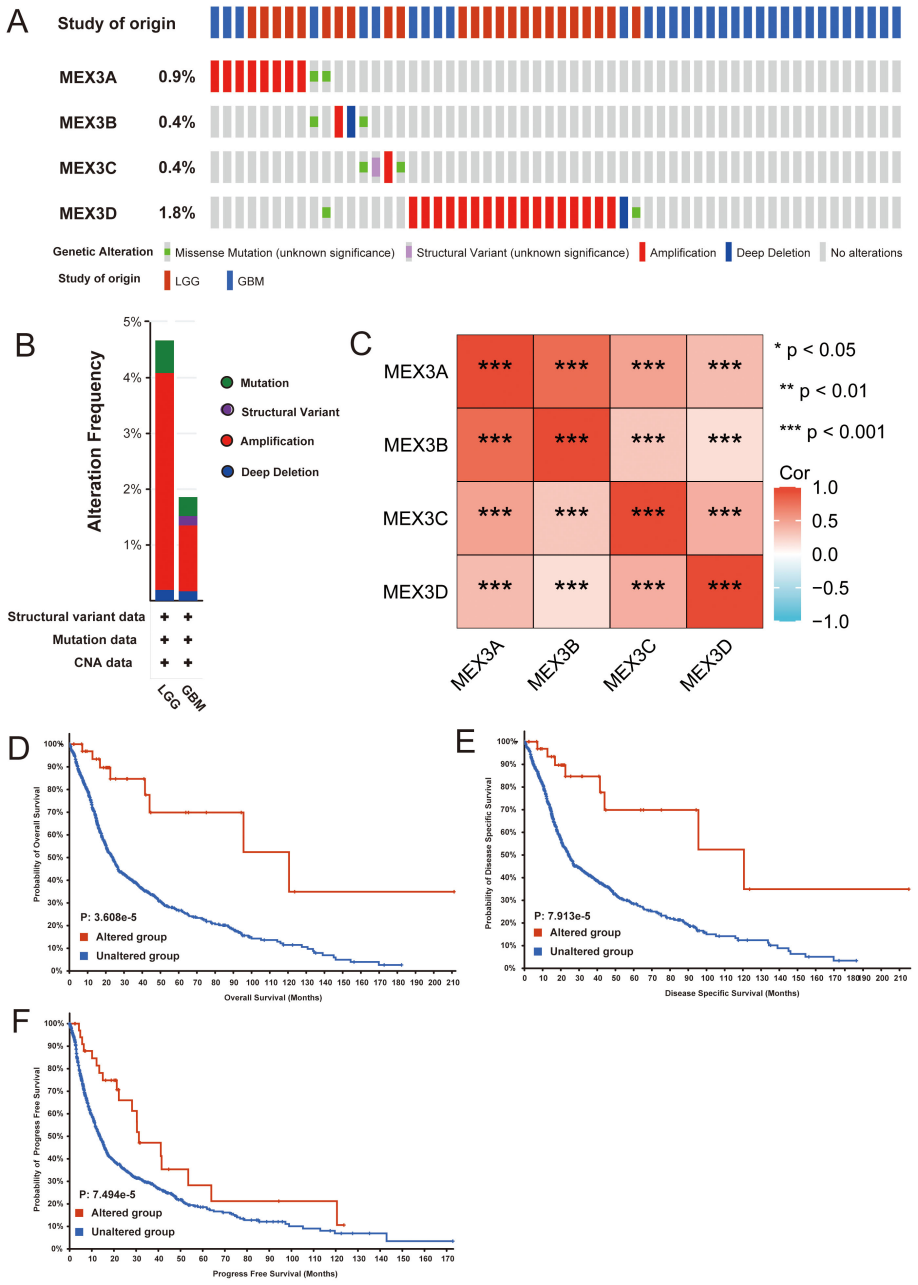
Genetic alterations in MEX3 family genes and their relationship with prognosis of glioma patients. (*p< 0.05, **p< 0.01, ***p< 0.001). **(A, B)** Summary of alterations in different expressed MEX3 families genes in glioma. **(C)** Correlations of MEX3 family genes with each other in glioma. **(D–F)** Genetic alterations in MEX3 family genes were associated with longer overall survival (OS) **(D)**, disease-specific survival (DSS) **(E)**, and progress-free interval (PFI) **(F)** of glioma patients.

Epigenetic modifications are crucial in the initial phases of tumor development. To investigate genetic alterations in the MEX3 gene family within glioma, we utilized the cBioPortal online platform, focusing on mutations and copy number variations. The specific genetic changes, along with their corresponding alteration frequencies, are depicted in **Figure 2A**. Among the MEX3 genes, MEX3D exhibited the highest mutation rate (1.8%), with amplifications being the most frequent alteration type. MEX3A presented both missense mutations and amplifications (0.9%), while MEX3B displayed missense mutations, amplifications, and deep deletions (0.9%). MEX3C, in contrast, harbored missense mutations, structural variations, and amplifications (0.4%). The genetic modifications affecting MEX3 genes were diverse, influencing 4.67% of 514 patients diagnosed with LGG and 1.86% of 592 patients with GBM, as illustrated in **Figure 2B**. To further understand the relationships among MEX3 gene family members, we performed a Pearson correlation analysis. The findings, presented in **Figure 2C**, indicate significant positive correlations between MEX3A, MEX3B, MEX3C, and MEX3D. Additionally, we examined the association between genetic alterations in the MEX3 gene family and key clinical outcomes, including overall survival (OS), disease-specific survival (DSS), and progression-free survival (PFS) in glioma patients. Kaplan-Meier survival analysis, coupled with a log-rank test, revealed a statistically significant correlation between MEX3 genetic variations and prolonged OS (P = 3.608e-5), DSS (P = 7.913e-5), and PFS (P = 7.494e-5) (**Figures 2D–F**). These findings indicate that genetic alterations in MEX3 genes could play an essential role in glioma prognosis.

**Figure 3:**
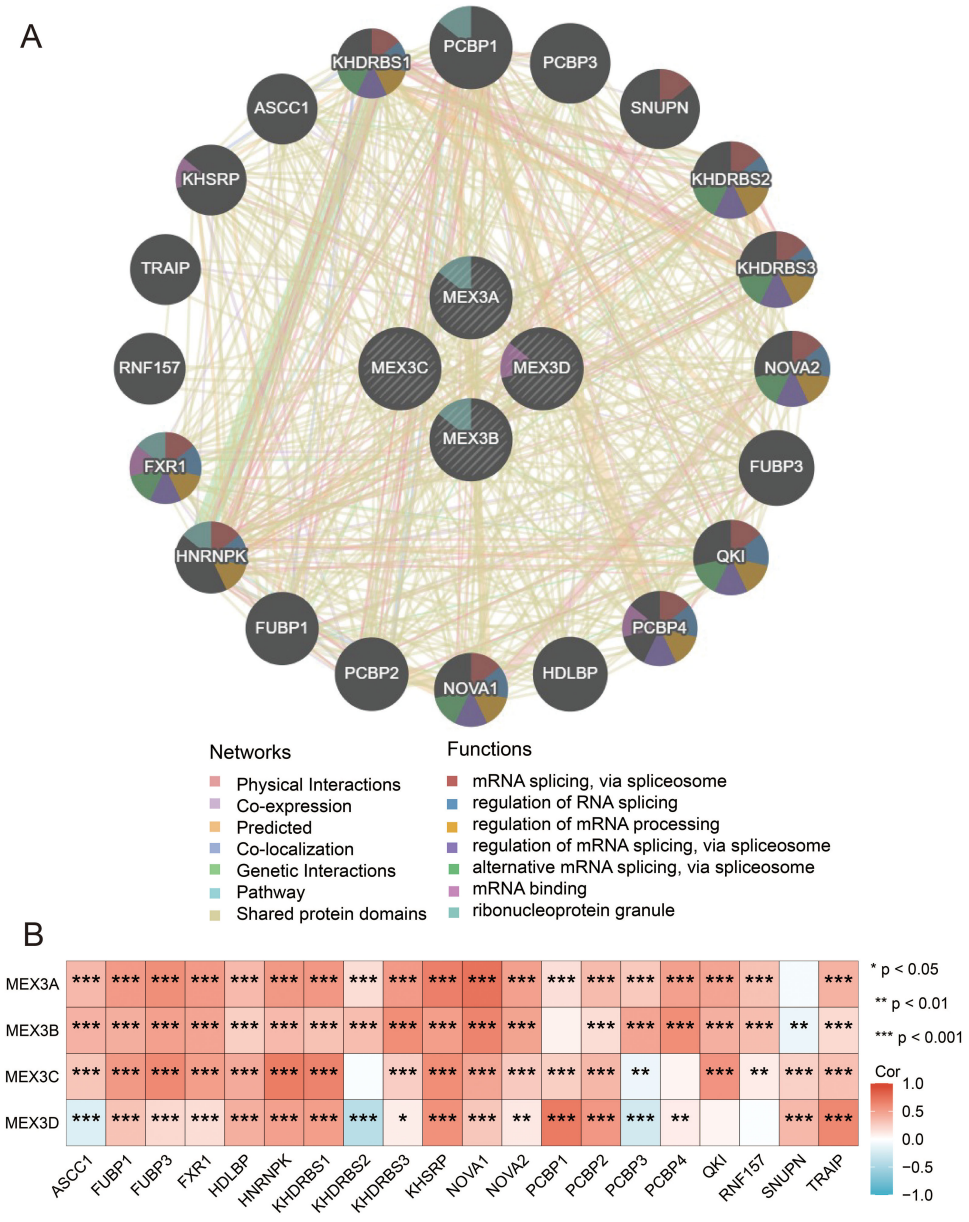
Protein-protein interaction (PPI) network of MEX3 family genes. (*p< 0.05, **p< 0.01, ***p< 0.001). **(A)** A protein-protein interaction (PPI) network was constructed by GeneMANIA. **(B)** The relationships between the enrichment of the 20 significant interactors and MEX3 family genes in glioma.

To systematically identify potential functional partners of the MEX3 family, we utilized GeneMANIA to construct a PPI network. Our analysis identified 20 significant interactors: ASCC1, FUBP1, FUBP3, FXR1, HDLBP, HNRNPK, KHDRBS1, KHDRBS2, KHDRBS3, KHSRP, NOVA1, NOVA2, PCBP1, PCBP2, PCBP3, PCBP4, QKI, RNF157, SNUPN, and TRAIP (**Figure 3A**). Functional enrichment analysis demonstrated that these interacting partners, together with MEX3 family members, are primarily involved in key biological processes including: mRNA splicing via spliceosome, Regulation of RNA splicing, Regulation of mRNA processing, Alternative mRNA splicing via spliceosome, Ribonucleoprotein granule formation, and mRNA binding. To further investigate the relationships between MEX3 family genes and these 20 significant interactors, we performed comprehensive correlation analyses of their expression patterns in glioma. As shown in **Figure 3B**, we observed statistically significant correlations (P< 0.05) between MEX3 family genes and several interactors including ASCC1, FUBP1, FUBP3, FXR1, HDLBP, HNRNPK, KHDRBS1, KHDRBS3, KHSRP, NOVA1, NOVA2, PCBP2, PCBP3, and TRAIP in glioma.

### 3.3 Immune correlation

**Figure 4:**
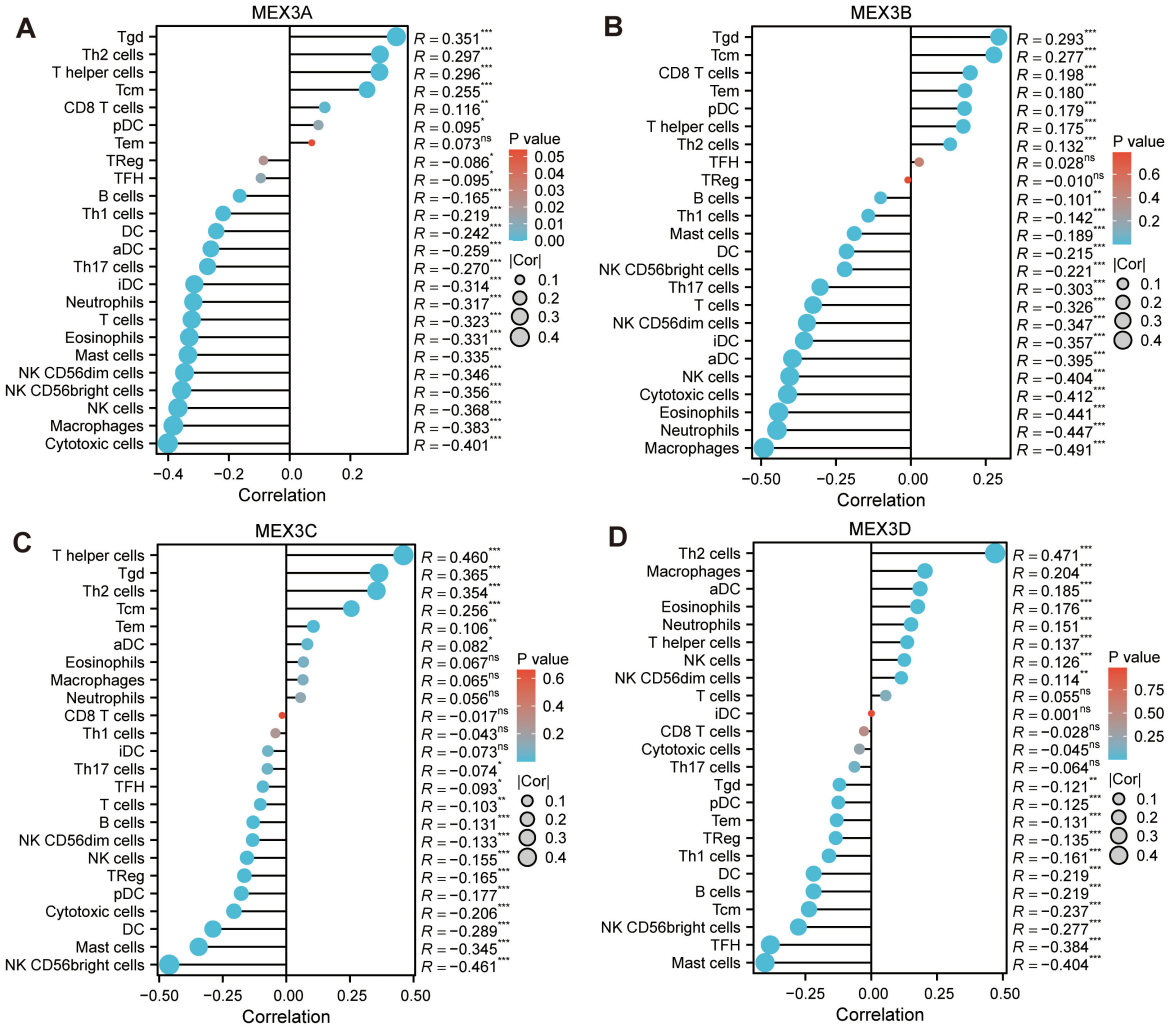
MEX3 family genes expressions and tumor immunity. In the bar graph, MEX3A **(A)**, MEX3B **(B)**, MEX3C **(C)** and MEX3D **(D)** expressions were related to 24 immune infiltration cells of glioma. (*p< 0.05, **p< 0.01, ***p< 0.001).

As illustrated in **Figure 4**, the ssGSEA algorithm was employed to assess the associations between the relative abundance of 24 immune cell types and the expression levels of MEX3 family genes in glioma (sample sizes provided in **Supplementary Table 10**). MEX3A expression exhibited a significant positive correlation with Tgd, Th2 cells, T helper cells, Tcm, CD8 T cells, and pDC. Conversely, a significant negative correlation was observed between MEX3A expression and multiple immune cells, including TReg, TFH, B cells, Th1 cells, DC, aDC, Th17 cells, iDC, Neutrophils, T cells, Eosinophils, Mast cells, NK CD56dim cells, NK CD56bright cells, NK cells, Macrophages, and Cytotoxic cells (**Figure 4A**). Similarly, as depicted in **Figure 4B**, seven immune cell types-Tgd, Tcm, CD8 T cells, Tem, pDC, T helper cells, and Th2 cells-showed a positive correlation with MEX3B expression. In contrast, MEX3B expression was negatively correlated with 15 immune cell types, including B cells, Th1 cells, Mast cells, DC, NK CD56bright cells, Th17 cells, T cells, NK CD56dim cells, iDC, aDC, NK cells, Cytotoxic cells, Eosinophils, Neutrophils, and Macrophages. Regarding MEX3C, its expression was significantly positively correlated with T helper cells, Tgd, Th2 cells, Tcm, and aDC, whereas a negative association was detected between MEX3C expression and Th17 cells, TFH, T cells, B cells, NK CD56dim cells, NK cells, TReg, pDC, Cytotoxic cells, DC, Mast cells, and NK CD56bright cells (**Figure 4C**). Meanwhile, MEX3D expression demonstrated a positive correlation with eight immune cell types, including Th2 cells, Macrophages, aDC, Eosinophils, Neutrophils, T helper cells, NK cells, and NK CD56dim cells. However, a significant negative correlation was identified between MEX3D expression and 11 immune cell types, namely Tgd, pDC, Tem, TReg, Th1 cells, DC, B cells, Tcm, NK CD56bright cells, TFH, and Mast cells (**Figure 4D**).

**Figure 5:**
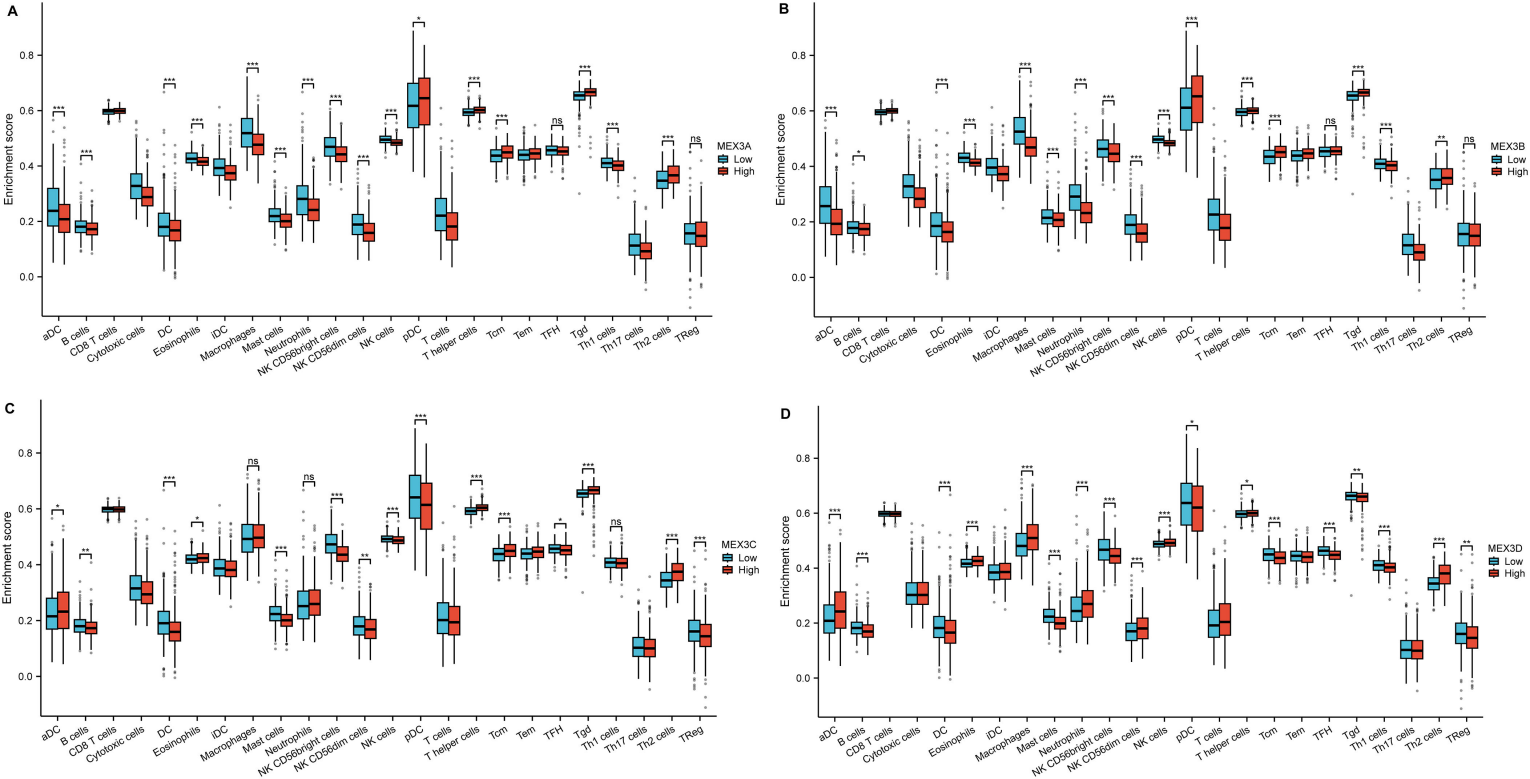
The relationships between the enrichment of immune cells and MEX3 family genes **(A-D)** high and low expression groups of glioma. (*p< 0.05, **p< 0.01, ***p< 0.001.).

To further analyze immune cell enrichment in glioma patients with high and low expression of MEX3 genes, the Wilcoxon rank-sum test was conducted (**Figure 5**, sample sizes provided in **Supplementary Table 11**). The results indicated that, compared to the MEX3A low-expression group, the high-expression group exhibited a greater abundance of pDC, T helper cells, Tcm, Tgd, and Th2 cells. In contrast, aDC, B cells, DC, Eosinophils, Macrophages, Mast cells, Neutrophils, NK CD56bright cells, NK CD56dim cells, NK cells, and Th1 cells were less enriched in the MEX3A high-expression group (**Figure 5A**). Similarly, compared to the MEX3B low-expression group, the MEX3B high-expression group demonstrated increased enrichment of NK cells, T helper cells, Tcm, Tgd, and Th2 cells, whereas the levels of aDC, B cells, Cytotoxic cells, DC, Macrophages, Mast cells, Neutrophils, NK CD56bright cells, NK CD56dim cells, NK cells, and Th1 cells were reduced (**Figure 5B**). For MEX3C, aDC, Eosinophils, T helper cells, Tcm, Tgd, and Th2 cells were significantly enriched in the high-expression group compared to the low-expression group. However, B cells, DC, Mast cells, NK CD56bright cells, NK CD56dim cells, NK cells, pDC, and TReg exhibited significantly lower enrichment levels in the MEX3C high-expression group (**Figure 5C**). Finally, in the MEX3D high-expression group, aDC, Eosinophils, Macrophages, Neutrophils, NK CD56dim cells, T helper cells, and Th2 cells were significantly upregulated compared to the low-expression group. In contrast, B cells, DC, Mast cells, NK CD56bright cells, pDC, Tcm, TFH, Tgd, Th1 cells, and TReg showed significantly lower enrichment in the MEX3D high-expression group (**Figure 5D**).

**Figure 6:**
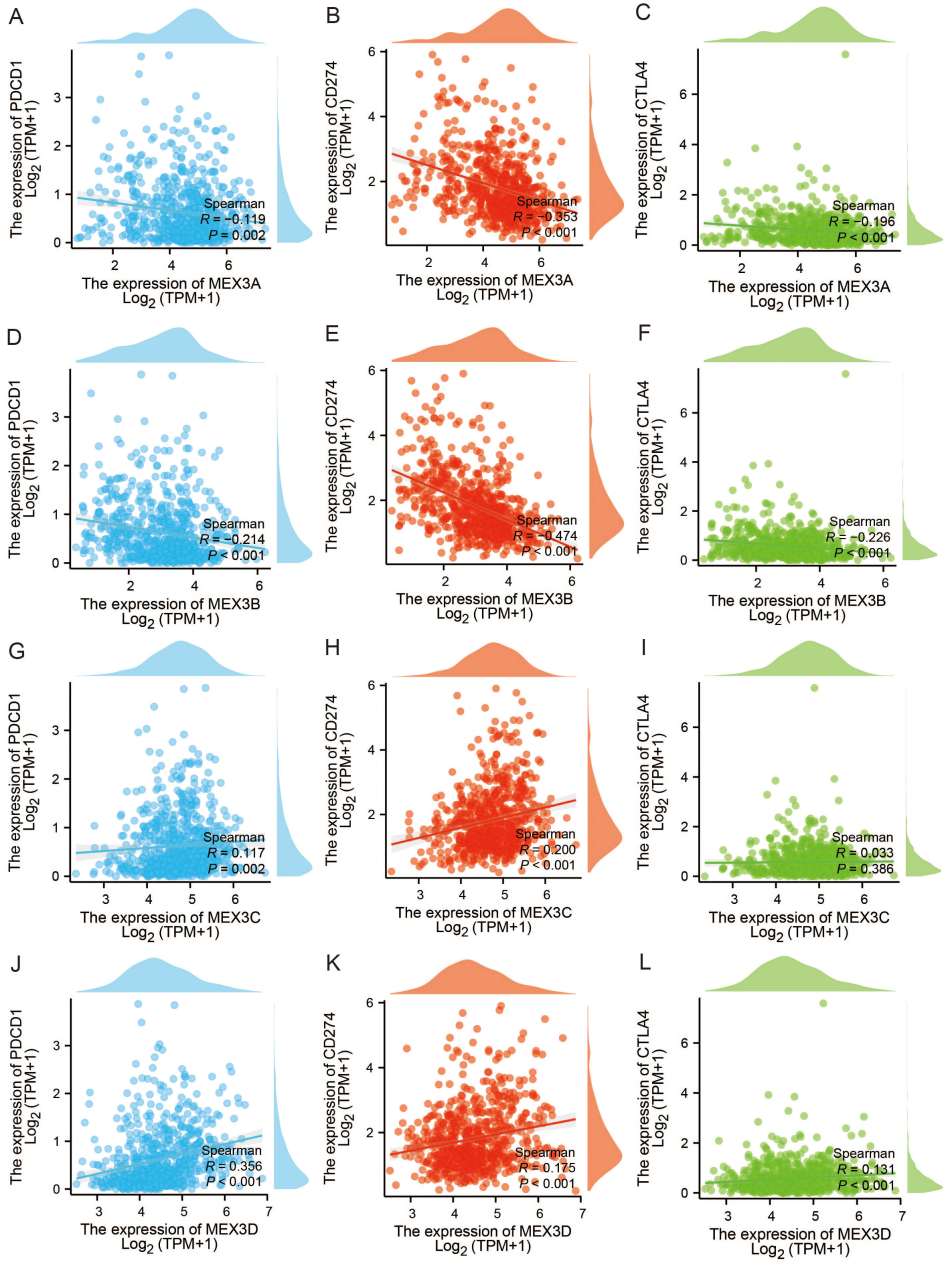
The relationships between immune checkpoint genes, including PD-1 (PDCD1), PD-L1 (CD274), and CTLA4, and MEX3 family genes of glioma. **(A–C)** The relationships between PDCD1 **(A)**, CD274 **(B)**, CTLA4 **(C)** and MEX3A in glioma. **(D-F)** The relationships between PDCD1 **(D)**, CD274 **(E)**, CTLA4 **(F)** and MEX3B in glioma. **(G-I)** The relationships between PDCD1 **(G)**, CD274 **(H)**, CTLA4 **(I)** and MEX3C in glioma. **(J–L)** The relationships between PDCD1 **(J)**, CD274 **(K)**, CTLA4 **(L)** and MEX3D in glioma.

To further explore the immunological significance of MEX3 family genes, we examined their associations with key immune checkpoint molecules, including PD-1 (PDCD1), PD-L1 (CD274), and CTLA4, in glioma. As shown in **Figures 6A–F**, MEX3A (**Figures 6A–C**) and MEX3B (**Figures 6D–F**) expression levels displayed a significant negative correlation with PDCD1, CD274, and CTLA4. In contrast, MEX3C expression demonstrated a significant positive correlation with PDCD1 and CD274 (**Figures 6G, H**), though no statistically significant association was found between MEX3C and CTLA4 (**Figure 6I**). Meanwhile, MEX3D expression exhibited strong positive correlations with all three immune checkpoint genes-PDCD1, CD274, and CTLA4 (**Figures 6J–L**).

### 3.4 Pathway and functional enrichment analyses

**Figure 7:**
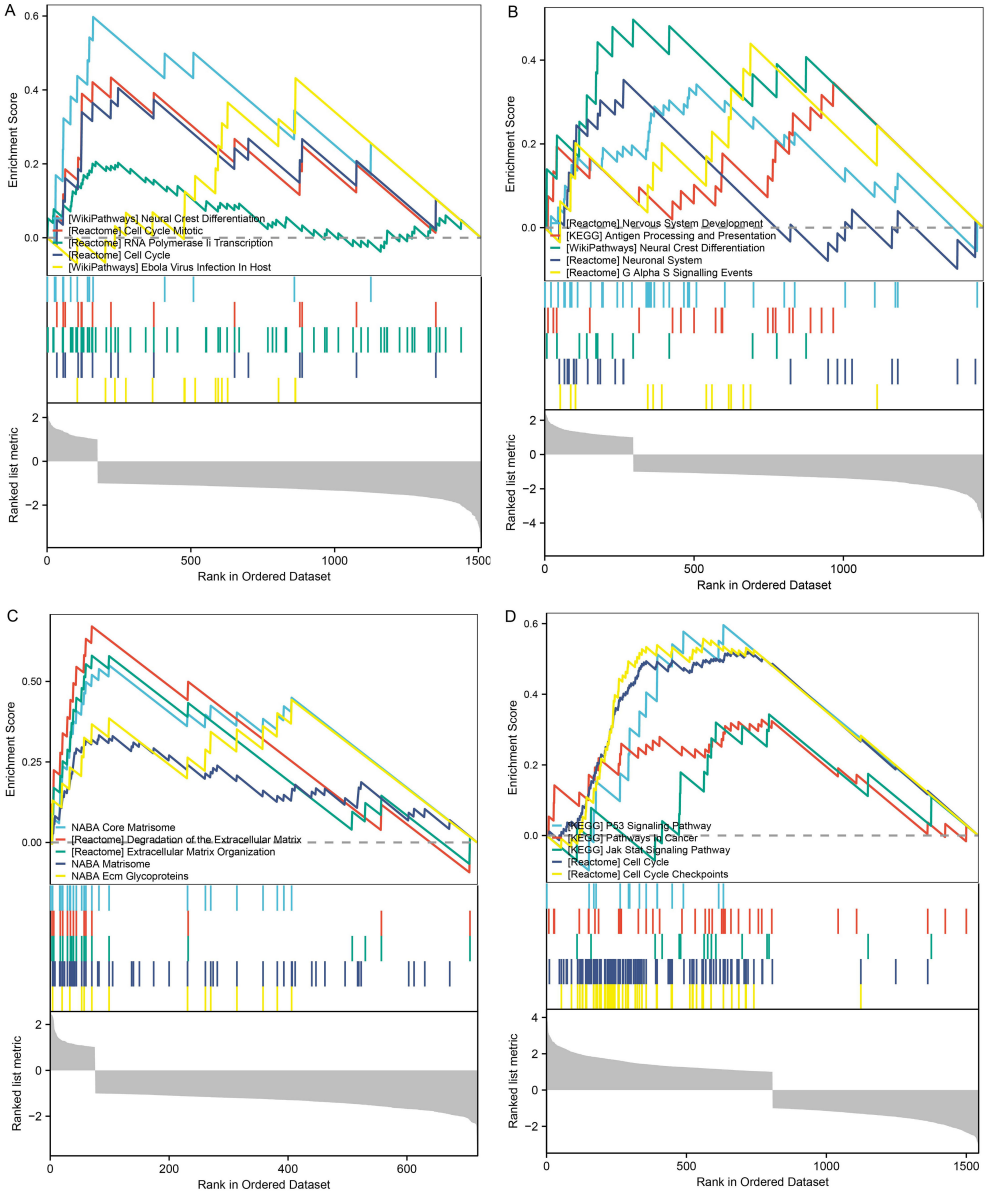
Gene set enrichment analysis (GSEA) of MEX3 family genes in glioma. GESA was performed to explore the molecular mechanisms mediated by MEX3A **(A)**, MEX3B **(B)**, MEX3C **(C)** and MEX3D **(D)** in glioma.

To explore the molecular mechanisms mediated by MEX3 family genes in glioma, we performed gene set enrichment analysis (GSEA) (**Figure 7**). The results revealed that MEX3A expression was significantly associated with pathways including Neural Crest Differentiation, Cell Cycle Mitotic, RNA Polymerase II Transcription, Cell Cycle, and Ebola Virus Infection in Host (**Figure 7A**). MEX3B expression was enriched in Nervous System Development, Antigen Processing and Presentation, Neural Crest Differentiation, Neuronal System, and G Alpha S Signaling Events (**Figure 7B**). MEX3C expression was significantly linked to Core Matrisome, Degradation of the Extracellular Matrix, Extracellular Matrix Organization, Matrisome, and ECM Glycoproteins (**Figure 7C**). MEX3D expression was associated with P53 Signaling Pathway, Pathways in Cancer, JAK-STAT Signaling Pathway, Cell Cycle, and Cell Cycle Checkpoints (**Figure 7D**).

**Figure 8:**
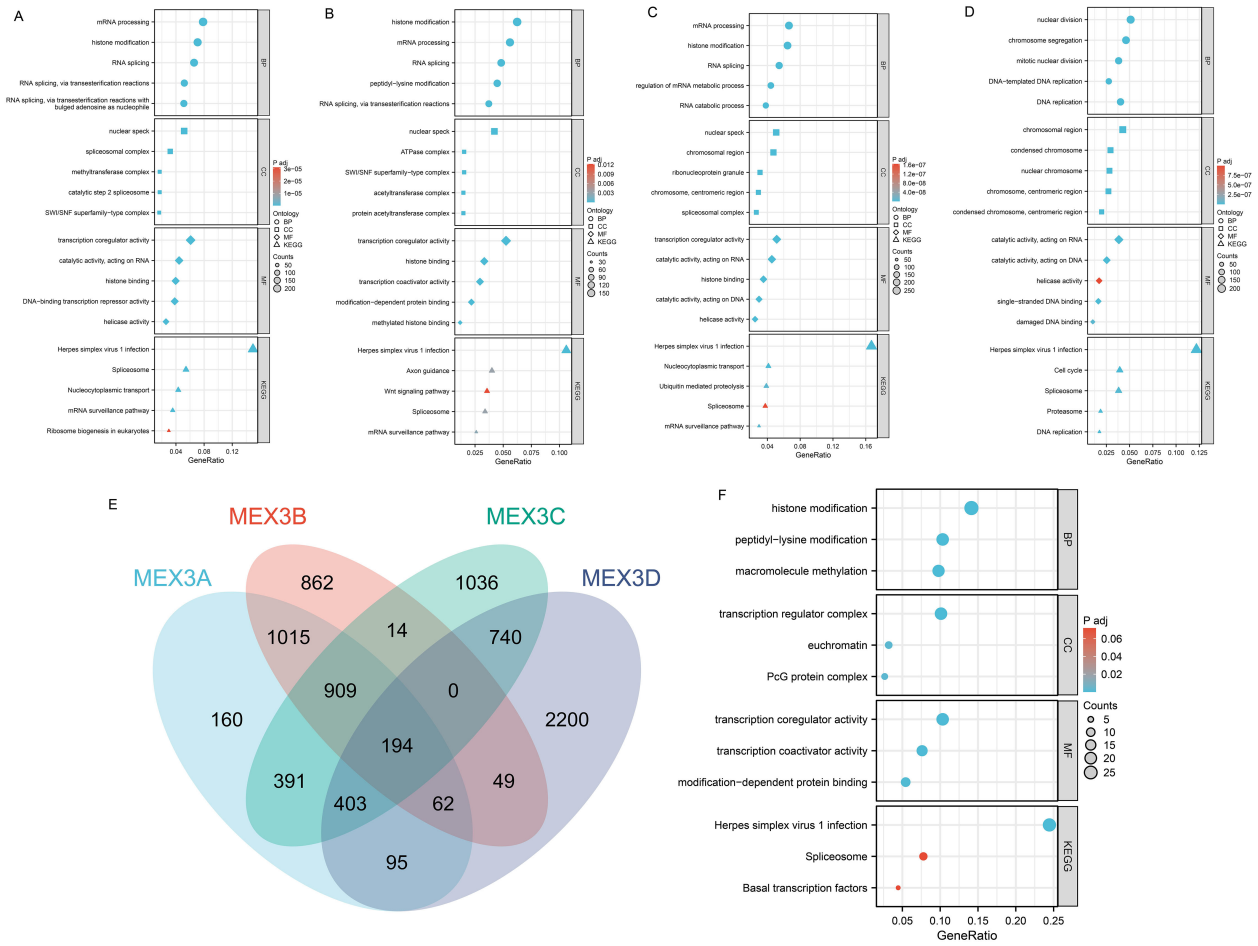
Gene Ontology (GO) and Kyoto Encyclopedia of Genes and Genomes (KEGG) enrichment analyses on the correlated genes of MEX3A, MEX3B, MEX3C and MEX3D (|R| > 0.4, P< 0.001) in glioma, and Genes co-expressed with MEX3 family in glioma and enrichment analysis of MEX3 family co-expression genes in glioma. **(A–D)** GO and KEGG enrichment analyses of MEX3A **(A)**, MEX3B **(B)**, MEX3C **(C)** and MEX3D **(D)** correlated genes in glioma. Bubble charts of GO and KEGG terms. **(E)** A Venn diagram illustrates 194 intersecting genes co-expressed with the MEX3 family genes in glioma (|R| > 0.4, P< 0.001). **(F)** Go and KEGG enrichment analyses of MEX3 family genes and their co-expression genes in glioma. Bubble charts of GO and KEGG terms.

In the TCGA transcriptome dataset, the number of genes exhibiting a positive correlation with MEX3A, MEX3B, MEX3C, and MEX3D was 8,759, 8,326, 10,815, and 10,595, respectively. Conversely, the number of genes negatively correlated with MEX3, MEX3L1, MEX3L2, and MEX3L3 was 3,535, 4,115, 1,553, and 2,746, respectively (sample sizes detailed in **Supplementary Table 12**). **Supplementary Figure 3** presents the ten most positively correlated and ten most negatively correlated genes for each MEX3 family member (sample sizes detailed in **Supplementary Table 13**). To gain further insight into the biological functions of MEX3 family genes in glioma, we performed Gene Ontology (GO) and Kyoto Encyclopedia of Genes and Genomes (KEGG) enrichment analyses on the correlated genes (|R| > 0.4, P< 0.001) (**Figure 8**). The GO analysis classified these genes into three major categories: molecular functions (MF), cellular components (CC), and biological processes (BP). For MEX3A, the most significantly enriched biological processes (BPs) included mRNA processing, histone modification, and various forms of RNA splicing, particularly those involving transesterification reactions. In the cellular component (CC) category, MEX3A was associated with the nuclear speck, spliceosomal complex, methyltransferase complex, catalytic step 2 spliceosome, and SWI/SNF superfamily-type complex. Enriched molecular functions (MFs) included transcription coregulator activity, catalytic activity acting on RNA, histone binding, DNA-binding transcription repressor activity, and helicase activity. KEGG pathway analysis indicated involvement in Herpes simplex virus 1 infection, Spliceosome, Nucleocytoplasmic transport, mRNA surveillance pathway, and Ribosome biogenesis in eukaryotes (**Figure 8A**). For MEX3B, enriched BPs included histone modification, mRNA processing, RNA splicing, and peptidyl-lysine modification. Associated CC terms were nuclear speck, ATPase complex, SWI/SNF superfamily-type complex, acetyltransferase complex, and protein acetyltransferase complex. In terms of MFs, MEX3B was linked to transcription coregulator activity, histone binding, transcription coactivator activity, modification-dependent protein binding, and methylated histone binding. KEGG pathways included Herpes simplex virus 1 infection, Axon guidance, Wnt signaling pathway, Spliceosome, and mRNA surveillance pathway (**Figure 8B**). MEX3C was primarily associated with BPs such as mRNA processing, histone modification, RNA splicing, regulation of mRNA metabolic process, and RNA catabolic process. Enriched CCs included nuclear speck, chromosomal region, ribonucleoprotein granule, chromosome (centromeric region), and spliceosomal complex. Key MFs were transcription coregulator activity, catalytic activity acting on RNA and DNA, histone binding, and helicase activity. KEGG pathway analysis revealed involvement in Herpes simplex virus 1 infection, Nucleocytoplasmic transport, Ubiquitin-mediated proteolysis, Spliceosome, and mRNA surveillance pathway (**Figure 8C**). For MEX3D, enriched BPs included nuclear division, chromosome segregation, mitotic nuclear division, and DNA-templated DNA replication. Associated CCs included chromosomal region, condensed chromosome, nuclear chromosome, and centromeric region. MFs included catalytic activity acting on RNA and DNA, helicase activity, single-stranded DNA binding, and damaged DNA binding. KEGG analysis indicated enrichment in pathways such as Herpes simplex virus 1 infection, Cell cycle, Spliceosome, Proteasome, and DNA replication (**Figure 8D**). Additionally, a Venn diagram (**Figure 8E**) illustrates the 194 overlapping genes that are co-expressed with MEX3 family genes in glioma (|R| > 0.4, P< 0.001) (sample sizes available in **Supplementary Table 14**). To gain further insight into the biological functions of MEX3 family co-expressed genes in glioma, we also performed GO and KEGG enrichment analyses on the co-expressed genes. Among the primary BPs influenced by MEX3 family co-expressed genes were histone modification, peptidyl-lysine modification, and macromolecule methylation. In terms of CCs, transcription regulator complex, euchromatin, and the PcG protein complex were the most significantly associated. Regarding MFs, transcription coregulator activity, transcription coactivator activity, and modification-dependent protein binding were among the primary functional roles. KEGG pathway analysis further indicated that MEX3 family co-expressed genes were implicated in pathways related to herpes simplex virus 1 infection, spliceosome activity, and basal transcription factors (**Figure 8F**).

### 3.5 Clinical and therapeutic implications

**Figure 9:**
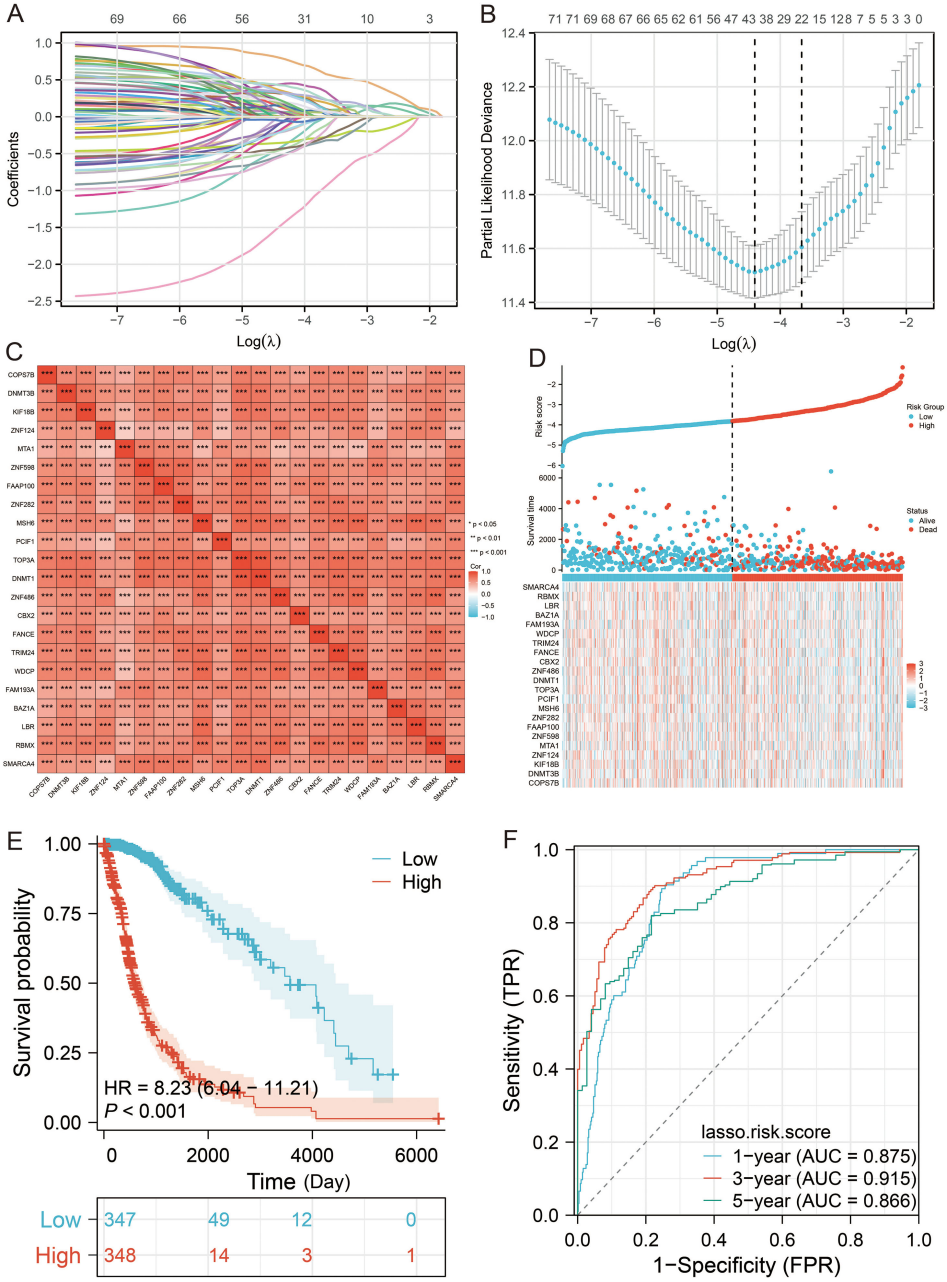
Development of a prognostic model based on MEX3 family co-expression Genes in glioma. (*p< 0.05, **p< 0.01, ***p< 0.001). **(A, B)** LASSO regression analysis was performed to identify 22 key prognostic genes. **(C)** Pearson correlation analysis revealed strong positive interrelationships among these 22 genes in glioma. **(D)** Risk score analysis of the 22 prognostic risk MEX3 family co-expression genes prognostic signature in glioma. **(E)** KM survival curves comparing overall survival between high- and low-risk groups in glioma. **(F)** Time-dependent ROC curves evaluating the predictive accuracy of the risk score in glioma.

**Figure 10:**
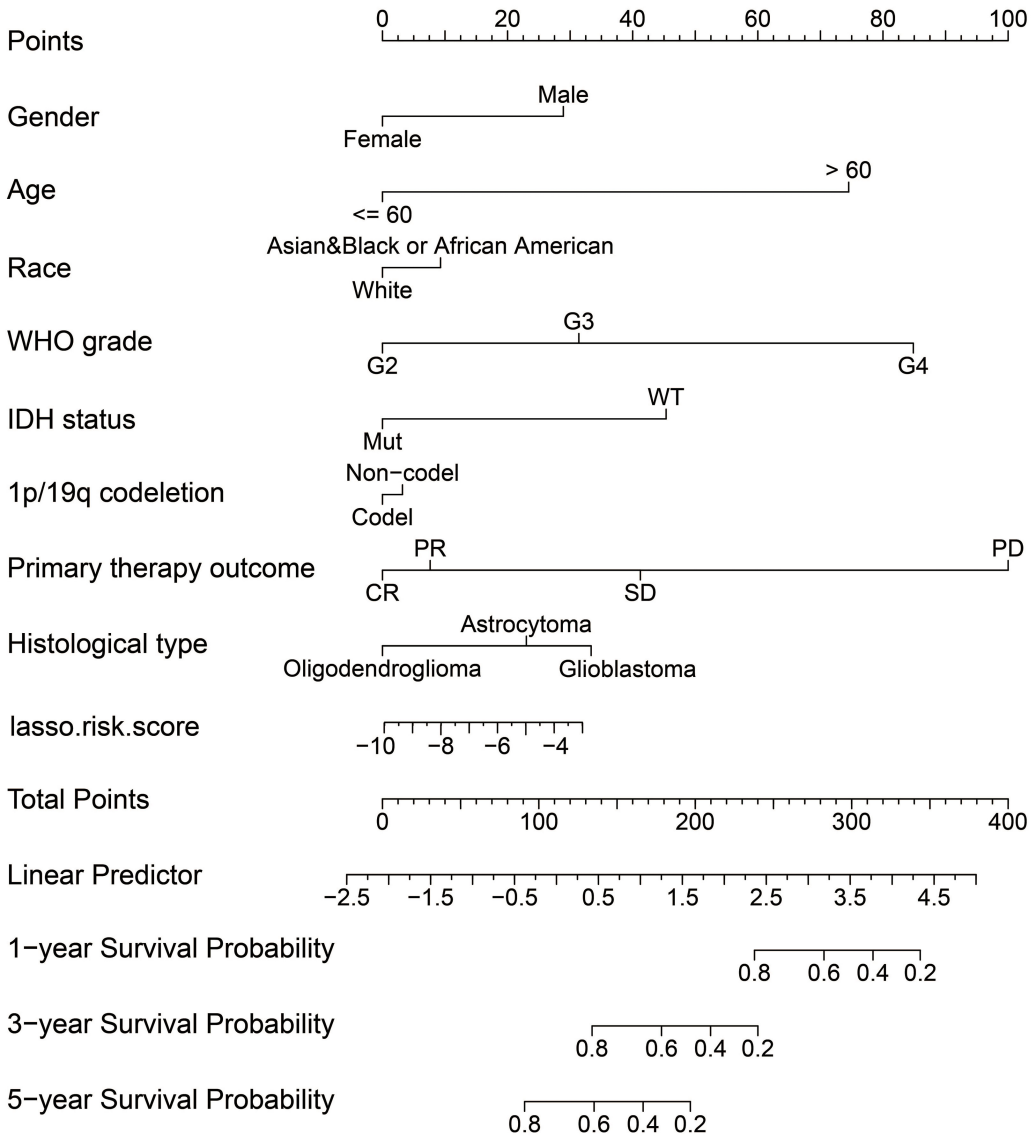
Development of a nomogram for predicting survival probability in glioma patients. The prognostic nomogram integrating the LASSO.risk.score with key pathological characteristics to predict survival outcomes in glioma patients.

Through systematic analysis, we first performed univariate Cox regression analysis, identifying 77 MEX3 family co-expression-related genes (including UBN1, PHTF2, and COPS7B) as potential prognostic biomarkers (**Supplementary Table 15**). Subsequent LASSO regression analysis refined this to 22 key prognostic genes: COPS7B, DNMT3B, KIF18B, ZNF124, MTA1, ZNF598, FAAP100, ZNF282, MSH6, PCIF1, TOP3A, DNMT1, ZNF486, CBX2, FANCE, TRIM24, WDCP, FAM193A, BAZ1A, LBR, RBMX, and SMARCA4 (**Figures 9A, B**). Pearson correlation analysis revealed strong positive interrelationships among these 22 genes in glioma (all P< 0.05; **Figure 9C**). Based on these findings, we constructed a prognostic model using the following risk score formula: Risk score = (COPS7B × 0.0546) + (DNMT3B × 0.5777) + (KIF18B× 0.09152) + (ZNF124 × −0.0007) + (MTA1× -0.2770) + (ZNF598 × 0.1039) + (FAAP100 × 0.0990) + (ZNF282 × 0.3402) + (MSH6 × 0.0361) + (PCIF1× -0.3198) + (TOP3A× 0.0718) + (DNMT1× 0.1897) + (ZNF486× 0.0448) + (CBX2× 0.0740) + (FANCE× -0.1173) + (TRIM24× 0.2283) + (WDCP× 0.2344) + (FAM193A× -0.2766) + (BAZ1A× -0.0011) + (LBR× -0.0004) + (RBMX× -0.1783) + (SMARCA4× -0.9400). To rigorously evaluate the prognostic performance of our MEX3 family co-expression gene signature in glioma, we implemented a comprehensive validation approach. The cohort of TCGA 699 patients was dichotomized into low-risk (n = 349) and high-risk (n = 350) groups using the median risk score as the cutoff threshold. Our analyses yielded three key findings: clinical outcome assessment demonstrated significantly worse prognosis in high-risk patients, manifesting as both elevated mortality rates and markedly shorter overall survival duration compared to low-risk patients (**Figure 9D**). Kaplan-Meier survival curves confirmed the robust discriminative power of our model, with high-risk patients showing substantially poorer survival outcomes (P< 0.05; **Figure 9E**). Time-dependent ROC analysis validated the model’s excellent predictive accuracy across multiple timepoints, with AUC values of: 0.875 for 1-year survival, 0.915 for 3-year survival, and 0.866 for 5-year survival (**Figure 9F**). To further evaluate the robustness of the risk model, its predictive performance was assessed in independent CGGA cohorts. In the CGGA_325 cohort, Kaplan-Meier survival analysis revealed a clear and statistically significant difference in overall survival between the high- and low-risk groups (**Supplementary Figure 4A**). Time-dependent ROC analysis demonstrated moderate prognostic performance, with area under the AUC values of 0.700, 0.747, and 0.754 for 1-, 3-, and 5-year survival, respectively (**Supplementary Figure 4B**). In the CGGA_693 cohort, Kaplan-Meier analysis similarly showed a significant separation of survival outcomes between the high- and low-risk groups (**Supplementary Figure 4C**), indicating preserved risk stratification capability. Although the time-dependent ROC curves indicated relatively modest predictive accuracy, the AUC values of 0.529, 0.608, and 0.623 for 1-, 3-, and 5-year survival, respectively, remained consistent with a trend toward prognostic discrimination (**Supplementary Figure 4D**). To enhance clinical applicability, we developed a comprehensive nomogram incorporating both molecular and clinical parameters (**Figure 10**). Notably, the LASSO.risk.score emerged as one of the most significant prognostic factors among various clinical variables.

**Figure 11:**
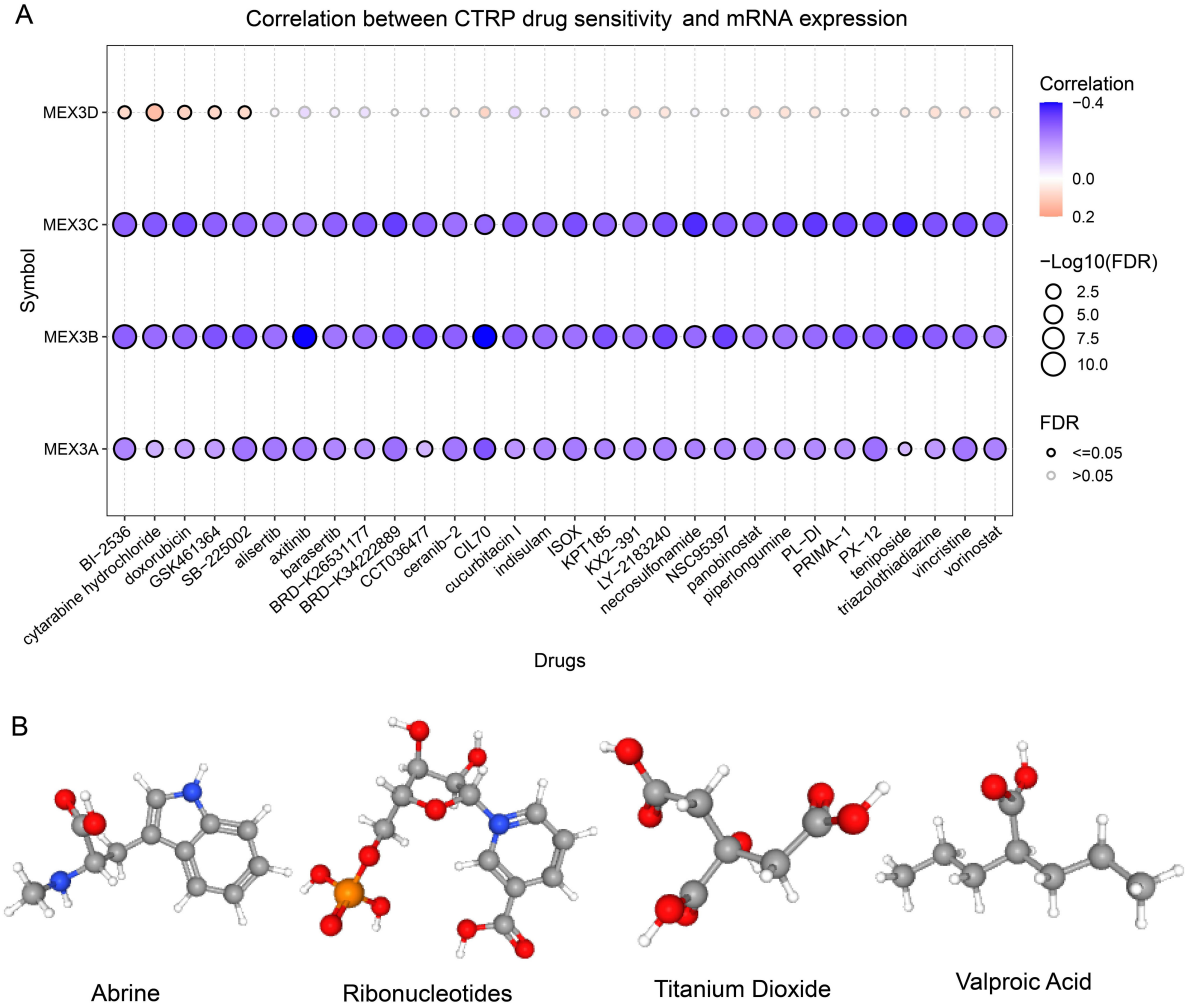
Correlation between MEX3 family gene expression and drug sensitivity. **(A)** Correlation between MEX3 family gene expression and drug sensitivity in cancer cell lines based on CTRP data. The heatmap displays Pearson correlation coefficients between mRNA expression levels of MEX3A, MEX3B, MEX3C, and MEX3D (rows) and the IC50 values of various compounds (columns). **(B)** Four kinds of MEX3-associated small-molecule compounds via CTD.

To investigate the potential role of MEX3 family genes in drug response, we analyzed the correlation between their expression levels and drug sensitivity (IC50 values) using data from the Cancer Therapeutics Response Portal (CTRP). As shown in **Figure 11A**, MEX3A, MEX3B, and MEX3C expression levels were significantly negatively correlated with sensitivity to a broad range of chemotherapeutic and targeted agents, while MEX3D showed limited associations. Specifically, MEX3B exhibited the strongest associations, showing high negative correlations with multiple drugs such as axitinib (cor = -0.398, FDR = 2.2E-28), CIL70 (cor = -0.399, FDR = 4.5E-14), and LY-2183240 (cor = -0.318, FDR = 1.35E-18). MEX3C showed similar patterns, with significant negative correlations with PL-DI (cor = -0.341, FDR = 8.8E-21), PRIMA-1, PX-12, and panobinostat. MEX3A was moderately correlated with sensitivity to agents including BRD-K34222889, SB-225002, and vincristine (|cor| > 0.23, FDR< 1E-10). In contrast, MEX3D showed only weak or non-significant correlations with most drugs, with few exceptions such as cytarabine hydrochloride (cor = 0.139, FDR = 0.0002) and narciclasine (cor = 0.134, FDR = 0.0005), suggesting a distinct pharmacogenomic profile. These results suggest that MEX3A-C genes may serve as potential biomarkers for chemosensitivity, whereas MEX3D may play a limited role in modulating drug response. We then utilized the Comparative Toxicogenomics Database (CTD) to identify seven small-molecule compounds that were commonly associated with all four MEX3 family members (MEX3A, MEX3B, MEX3C, and MEX3D). Notably, four of these compounds-Valproic Acid, titanium dioxide, abrine, and ribonucleotides-have been previously reported to possess anticancer properties (**Figure 11B**), suggesting their potential relevance in MEX3-related regulatory pathways and therapeutic applications.

### 3.6 Experimental validation

**Figure 12:**
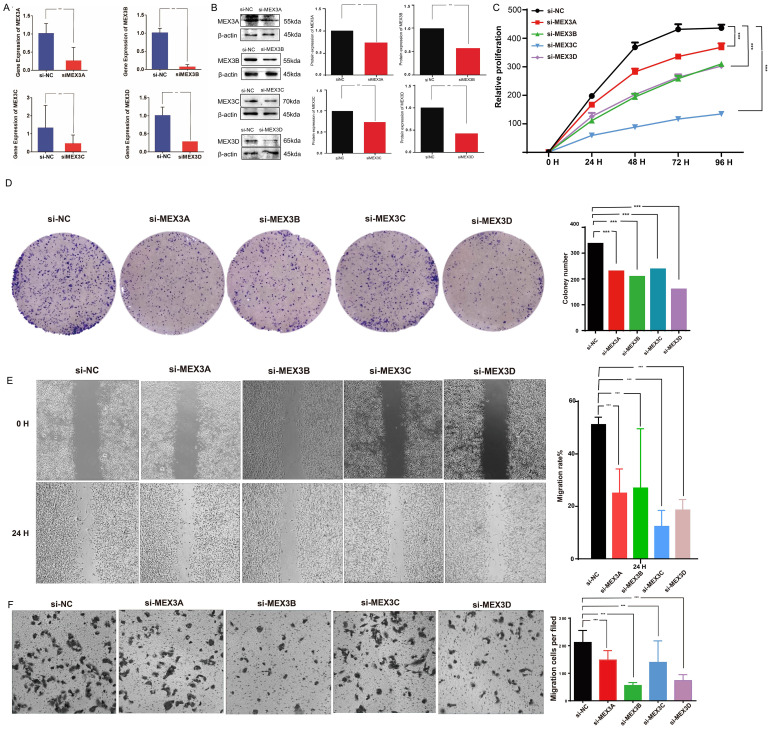
Functional validation of MEX3 family gene silencing in U251 glioma cells (***p< 0.001). **(A, B)** qRT-PCR and Western blot analyses confirmed that transfection with si-MEX3A, si-MEX3B, si-MEX3C, or si-MEX3D markedly reduced the corresponding mRNA and protein levels compared with the si-NC group, indicating effective gene knockdown. **(C, D)** Cell proliferation assays showed that silencing individual MEX3 family members significantly inhibited U251 cell growth. **(E, F)** Wound-healing and Transwell assays demonstrated that depletion of MEX3 genes markedly impaired the migratory and invasive capacities of U251 cells.

**Figure 13:**
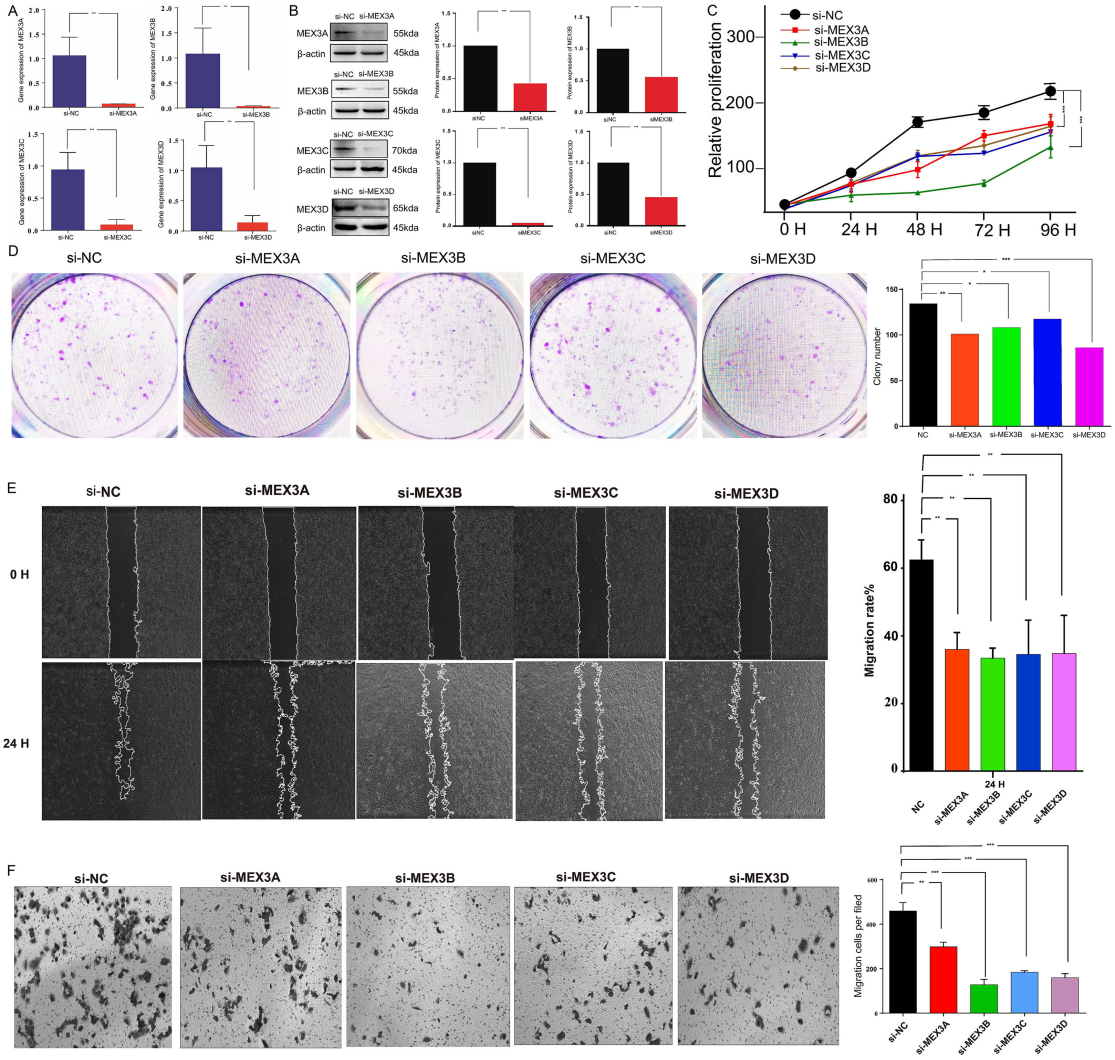
Effects of MEX3 family gene silencing on malignant phenotypes in LN229 glioma cells (*p< 0.05; **p< 0.01; ***p< 0.001). **(A, B)** qRT-PCR and Western blot analyses confirmed efficient knockdown of MEX3A, MEX3B, MEX3C, and MEX3D at the mRNA and protein levels, respectively. **(C, D)** Cell proliferation assays showed that silencing individual MEX3 family members significantly inhibited LN229 cell growth. **(E, F)** Wound-healing and Transwell assays demonstrated that depletion of MEX3 genes markedly reduced the migratory and invasive capacities of LN229 cells.

Furthermore, the expression levels of MEX3A, MEX3B, MEX3C, and MEX3D in transfected U251 glioma cells were assessed by qRT-PCR (**Figure 12A**) and Western blot analyses (**Figure 12B**). Compared with the si-NC group, both mRNA and protein levels of the corresponding MEX3 family members were markedly reduced following transfection with si-MEX3A, si-MEX3B, si-MEX3C, or si-MEX3D, confirming the efficiency of gene silencing. To further validate these findings, the effects of MEX3 family gene depletion on the malignant phenotypes of glioma cells were evaluated in U251 cells. Silencing individual MEX3 members significantly suppressed cell proliferation (**Figures 12C, D**) and markedly attenuated migratory and invasive abilities, as evidenced by wound-healing and Transwell assays (**Figures 12E, F**). Collectively, these results suggest that MEX3 family genes contribute to the proliferative, migratory, and invasive properties of glioma cells. Consistent results were observed in LN229 glioma cells, in which efficient knockdown of MEX3A, MEX3B, MEX3C, and MEX3D was confirmed at both the mRNA (**Figure 13A**) and protein levels (**Figure 13B**). Functional assays further demonstrated that silencing individual MEX3 family members significantly inhibited cell proliferation (**Figures 13C, D**) and reduced migratory and invasive abilities, as assessed by wound-healing and Transwell assays (**Figures 13E, F**). These findings further support a role for MEX3 family genes in the regulation of malignant phenotypes in glioma cells.

## 4 Discussion

Glioma, as the most prevalent and aggressive primary brain tumors, exhibits complex molecular heterogeneity (30). In this study, we performed an integrative analysis combining multi-omics bioinformatics profiling with *in vitro* functional assays to systematically characterize the expression patterns, prognostic significance, immune associations, and biological functions of the MEX3 family genes in glioma. Our results consistently demonstrate that MEX3 family members are aberrantly expressed in glioma and are closely associated with patient survival, malignant phenotypes, and features of the tumor immune microenvironment.

We first demonstrated that all four MEX3 genes are significantly upregulated in glioma tissues compared with normal brain samples, a finding consistently validated across independent datasets (TCGA, CGGA, and GSE16011). ROC curve analyses further supported their strong diagnostic potential, particularly for MEX3A and MEX3C, which exhibited excellent discriminatory power. These results are consistent with prior studies showing that MEX3 proteins act as RNA−binding regulators involved in post−transcriptional control and tumorigenesis, reinforcing the biological relevance of the MEX3 family in glioma (6, 31).

At the expression and prognostic level, MEX3A-D exhibited heterogeneous yet biologically meaningful patterns across glioma grades, molecular subtypes, and clinical outcomes. Notably, elevated expression of MEX3C and MEX3D was associated with unfavorable survival, whereas MEX3A and MEX3B showed comparatively more context-dependent or less aggressive associations. Multivariate Cox regression analyses further revealed that MEX3D remained independently associated with poor overall survival after adjustment for established clinical factors, including age, WHO grade, IDH mutation status, and primary therapy outcome. These findings suggest that, among the MEX3 family, MEX3D may represent a particularly robust prognostic indicator in glioma. Importantly, the robustness of these observations was strengthened through validation in multiple independent external cohorts. Consistent expression trends and prognostic stratification performance were confirmed in CGGA (mRNAseq_325 and mRNAseq_693) and GEO (GSE16011) datasets, which are derived from distinct patient populations and sequencing platforms. While the predictive accuracy of the risk model varied across cohorts, the overall preservation of prognostic discrimination supports the generalizability of our findings beyond a single dataset. Nevertheless, we acknowledge that prospective validation in dedicated clinical cohorts will be essential to further establish clinical utility.

The observed prognostic dichotomy among MEX3 family members likely reflects functional heterogeneity at the molecular level. All MEX3 proteins harbor both KH RNA−binding domains and a RING−type E3 ubiquitin ligase domain, enabling them to regulate gene expression through RNA stability, translation, and ubiquitin−mediated protein turnover (32). We therefore hypothesize that differential RNA target specificity, subcellular localization, or interaction partners may underlie the opposing clinical associations of individual MEX3 genes. Importantly, these mechanistic interpretations are currently based on correlative analyses and domain knowledge, and direct experimental validation remains necessary.

Epigenetic and genetic modifications are fundamental in glioma progression, affecting tumor growth, immune evasion, and treatment resistance (33). Genetic alteration analysis revealed relatively low but non−negligible mutation frequencies in MEX3 genes, with MEX3D showing the highest rate, predominantly driven by copy number amplification. Such amplifications may contribute to its elevated expression in glioblastoma and reinforce its potential oncogenic role. Interestingly, MEX3 alterations were more frequent in lower−grade gliomas than in GBM, suggesting that MEX3 dysregulation may occur early during glioma evolution, while later stages may be dominated by alternative oncogenic drivers or epigenetic mechanisms.

To elucidate the functional landscape of the MEX3 family in glioma, we constructed a PPI network using the GeneMANIA platform, revealing 20 proteins with strong interaction potential. These interactors-including FUBP1, HNRNPK, KHDRBS1-3, NOVA1/2, and PCBP family members-are predominantly RNA-binding proteins known to regulate diverse aspects of RNA metabolism, particularly alternative splicing, mRNA stabilization, and ribonucleoprotein complex assembly. Functional enrichment analysis indicated that MEX3 proteins and their interactors are significantly associated with mRNA splicing via spliceosome, regulation of mRNA processing, and ribonucleoprotein granule formation, suggesting that the MEX3 family may play a broader role in post-transcriptional regulation within glioma cells. Recent evidence supports this notion. For example, HNRNPK has been shown to contribute to glioma progression by regulating the splicing of genes involved in apoptosis and proliferation (34), while FUBP1 is frequently mutated in glioma, affecting transcriptional regulation and tumor metabolism (35). The observed correlations between MEX3 family members and these interactors in glioma samples further support functional co-regulation. Notably, strong positive correlations with KHDRBS1 and NOVA2, both well-established splicing regulators, reinforce the potential involvement of MEX3 proteins in modulating glioma-specific transcriptomic plasticity (36). Moreover, components such as FXR1 and PCBP2 have been implicated in glioma stemness and resistance to therapy through their regulation of mRNA stability and translation (37, 38). These findings suggest that MEX3 family proteins may interact with this regulatory axis to influence glioma malignancy. The enrichment in ribonucleoprotein granule formation also points to possible roles in stress granule dynamics, which are increasingly recognized as critical modulators of tumor cell survival under adverse microenvironmental conditions. These findings suggest that MEX3 proteins may contribute to glioma malignancy by coordinating RNA metabolism and transcriptomic plasticity, processes increasingly recognized as central to glioma heterogeneity and therapy resistance.

The tumor microenvironment (TME) plays a crucial role in glioma progression, influencing tumor growth, immune evasion, and therapeutic resistance (39). Our immune infiltration analyses revealed distinct immune landscapes associated with different MEX3 family members. In particular, MEX3D expression showed strong positive correlations with macrophage infiltration and immune checkpoint molecules, including PD−1, PD−L1, and CTLA−4. Importantly, these observations indicate association rather than causation. While our data suggest that high MEX3D expression is linked to an immunosuppressive microenvironment and T−cell exhaustion, we emphasize that any role of MEX3D in promoting M2 macrophage polarization or immune evasion remains hypothetical at this stage and warrants direct experimental investigation using functional and co−culture models. Tumor-associated macrophages (TAMs) in glioma are predominantly skewed toward an M2-like phenotype and represent a key driver of immunosuppression and tumor progression (40). Given that MEX3D is an RNA-binding protein with post-transcriptional regulatory capacity, it is plausible that elevated MEX3D expression may be linked to M2-associated immunosuppressive programs, although this hypothesis remains untested. Consistently, gene set enrichment analyses showed that high MEX3D expression was associated with immune- and cancer-related pathways, including p53 signaling, JAK-STAT signaling, and broad oncogenic pathways, all of which have been implicated in macrophage polarization and immune tolerance (41–43). However, these pathway-level associations do not establish a direct mechanistic role and should be interpreted as supportive context rather than causal evidence. Beyond macrophage-related features, we observed distinct correlations between MEX3 family members and immune checkpoint molecules. In particular, MEX3C and MEX3D showed positive associations with PD-1, PD-L1, and CTLA4, whereas MEX3A and MEX3B were negatively correlated with these checkpoints, suggesting divergent immune landscapes linked to different MEX3 members. Clinically, high MEX3D expression was further associated with higher TIDE scores and lower immunophenoscores, indicating a potentially immunosuppressive phenotype and reduced responsiveness to immune checkpoint blockade. MEX3D may potentially modulate immune checkpoint expression or contribute to immune evasion; however, the underlying mechanisms remain to be fully elucidated and warrant further investigation employing approaches such as co−culture assays, conditional knockout models, or interrogation of downstream signaling pathways. Our study follows an integrative research framework-combining large-scale bioinformatic screening with *in vitro* functional validation-that has been successfully applied in prior glioma biomarker studies, including investigations of voltage-gated sodium channel-related genes (44–46). Within this context, the observed correlations between MEX3D and immune checkpoint pathways should be viewed as hypothesis-generating rather than mechanistically conclusive. Future studies employing co-culture systems, conditional genetic models, and downstream signaling analyses will be essential to determine whether MEX3D plays an active regulatory role in immune checkpoint modulation or tumor immune escape.

Understanding the molecular networks involving the MEX3 family genes in glioma is critical for elucidating their biological functions and potential therapeutic implications. Our study identified thousands of genes that are positively and negatively correlated with MEX3 family members in glioma, with 8759, 8326, 10815, and 10595 genes positively correlated with MEX3A, MEX3B, MEX3C, and MEX3D, respectively. Additionally, GO and KEGG enrichment analyses provided insights into the functional pathways associated with MEX3 co-expressed genes, revealing potential roles in glioma progression. Genes co-expressed with MEX3 family members were significantly enriched in histone modification, peptidyl-lysine modification, and macromolecule methylation (6, 31). These findings suggest that MEX3 genes may play a role in epigenetic regulation, which is crucial for glioma development and progression. Previous studies have shown that epigenetic dysregulation, including histone and DNA methylation alterations, is a hallmark of gliomas (47). MEX3-associated genes were enriched in the transcription regulator complex, euchromatin, and PcG protein complex. This suggests a role for MEX3 family members in transcriptional regulation and chromatin remodeling, which are key processes in tumor initiation and maintenance. Polycomb group (PcG) proteins, in particular, are known to modulate gene silencing and promote cancer cell survival (48). Significant enrichment was observed in transcription coregulator activity, transcription coactivator activity, and modification-dependent protein binding. These findings align with previous reports that MEX3 proteins act as RNA-binding proteins (RBPs) involved in post-transcriptional regulation (6). RBPs contribute to mRNA stability, splicing, and translation, thereby influencing glioma cell survival, invasion, and therapy resistance (49). Additionally, KEGG pathway analysis identified significant enrichment in pathways related to herpes simplex virus 1 infection, spliceosome function, and basal transcription factors, suggesting a broader impact of MEX3 genes on glioma pathophysiology. Surprisingly, KEGG analysis revealed that MEX3 co-expressed genes were significantly enriched in the HSV-1 infection pathway. This may suggest a possible link between viral infections and glioma progression, as oncogenic viruses can modulate immune evasion and cell proliferation. Previous studies have indicated that viral infections, including cytomegalovirus (CMV) and HSV-1, might play a role in glioblastoma pathogenesis (50, 51). Aberrant splicing is a common feature in glioma, with dysregulated splicing factors contributing to tumor heterogeneity and therapy resistance (52). Enrichment in basal transcription factors suggests that MEX3 co-expressed genes might influence transcriptional control, further supporting their role in RNA regulation and gene expression modulation in glioma. Our study demonstrates that MEX3 family genes play critical roles in glioma by modulating transcriptional regulation, epigenetic modification, and RNA processing. GO and KEGG enrichment analyses revealed their involvement in pathways related to histone modification, spliceosome function, and viral infection, suggesting their potential as prognostic markers and therapeutic targets. Further experimental validation and functional studies are warranted to explore MEX3-targeted therapeutic strategies in glioma.

To extend the translational relevance of our findings, we developed a prognostic model based on MEX3 co−expression genes. The resulting signature demonstrated strong and independent prognostic performance and was further integrated into a nomogram to enhance clinical applicability. Notably, several components of the final signature-such as DNMT1, DNMT3B, MTA1, and SMARCA4-have previously been implicated in glioma pathogenesis and progression. DNA methyltransferases DNMT1 and DNMT3B are known epigenetic regulators frequently upregulated in glioma (53), contributing to genome-wide aberrant methylation and transcriptional silencing of tumor suppressor genes. SMARCA4, a core ATPase of the SWI/SNF chromatin remodeling complex, has also been reported to modulate glioma cell proliferation and stemness, with its loss linked to worse outcomes (54). Moreover, the involvement of MTA1, a metastasis-associated gene, underscores the model’s potential to reflect aggressive glioma phenotypes. Furthermore, while our prognostic model was validated in additional public datasets (CGGA), prospective validation in a dedicated, independent clinical cohort is warranted to further confirm its robustness and to evaluate its practical utility in a clinical setting.

The significant inverse correlations between MEX3A, MEX3B, and MEX3C expression and drug IC50 values suggest that these RNA-binding proteins may enhance cancer cell sensitivity to a wide range of anticancer agents. For instance, the strong negative correlation between MEX3B and axitinib-a VEGFR tyrosine kinase inhibitor-implies that MEX3B may modulate angiogenesis-related pathways, thereby influencing drug efficacy. Similarly, the sensitivity to Aurora kinase inhibitors such as alisertib and barasertib in high MEX3-expressing cells points toward a possible interaction with mitotic regulation mechanisms. Interestingly, MEX3D demonstrated little to no correlation with drug response, highlighting a divergent functional role within the family. The weak positive correlation with agents like cytarabine and doxorubicin may suggest involvement in DNA damage or repair-related pathways, albeit with limited biological impact. Collectively, these findings suggest that MEX3A-C could serve as predictive biomarkers for therapeutic response and potential candidates for combination therapy strategies aimed at enhancing drug sensitivity in cancer treatment. Further functional validation *in vitro* and *in vivo* is warranted to elucidate the mechanistic basis of these associations and their translational potential. The identification of small-molecule compounds targeting MEX3 family members provides novel insights into the therapeutic modulation of RNA-binding proteins in cancer. In our study, seven compounds were found to be commonly associated with MEX3A, MEX3B, MEX3C, and MEX3D based on data retrieved from the Comparative Toxicogenomics Database (CTD). Among them, Valproic Acid, titanium dioxide, abrine, and ribonucleotides have previously been documented to possess anticancer activities, underscoring their potential utility in targeting MEX3-driven oncogenic processes. Valproic Acid, a well-known histone deacetylase (HDAC) inhibitor, has demonstrated antiproliferative effects in various cancers through epigenetic reprogramming and apoptosis induction (55). Its association with all four MEX3 genes suggests that MEX3s may be involved in epigenetic pathways influenced by HDAC inhibition. Abrine, a plant-derived alkaloid, has been shown to induce apoptosis and inhibit proliferation in multiple tumor types via mitochondrial and caspase-dependent mechanisms (56). Titanium dioxide nanoparticles, while traditionally studied for drug delivery and imaging, have also been reported to generate reactive oxygen species and trigger oxidative stress-induced tumor cell death (57). Ribonucleotides, the building blocks of RNA, may impact RNA stability and translation dynamics, processes that are tightly regulated by RNA-binding proteins such as MEX3s (58). These findings highlight the possibility that pharmacologic targeting of MEX3-related pathways could be achieved through known or repurposed small molecules. However, functional experiments are needed to elucidate whether the observed compound–gene associations are direct or mediated through upstream regulatory networks. Further studies, including *in vitro* validation, molecular docking, and network pharmacology analysis, are warranted to explore the mechanistic underpinnings and therapeutic feasibility of these candidate compounds in MEX3-driven cancers.

The functional experiments provide direct evidence that MEX3 family genes contribute to malignant cellular behaviors in glioma. Using siRNA-mediated knockdown approaches, we demonstrated that depletion of MEX3A, MEX3B, MEX3C, or MEX3D significantly suppressed proliferation, migration, and invasion in both U251 and LN229 glioma cell lines. The inclusion of a second, widely used glioma cell model and confirmation at both mRNA and protein levels substantially enhance the credibility and generalizability of these experimental findings. Collectively, these results support a functional role of MEX3 family members in promoting aggressive glioma phenotypes.

Our study follows a methodological paradigm increasingly adopted in cancer research, in which large-scale bioinformatic screening is integrated with experimental validation to identify novel biomarkers and functional regulators. Similar strategies have been successfully applied in prior TCGA-based investigations of glioma and other cancers, reinforcing the validity of this approach. At the same time, we acknowledge the inherent limitations of bulk transcriptomic datasets, including sample heterogeneity, technical biases, and limited resolution of cell-type-specific effects (59–61). These factors necessitate cautious interpretation of differential expression and pathway enrichment results and further highlight the importance of independent validation.

This study has several limitations that should be acknowledged. Although integrated bioinformatic analyses and *in vitro* functional assays consistently associate MEX3 family genes with malignant progression and immune-related features in glioma, the precise molecular mechanisms-particularly whether and how MEX3 members regulate immune checkpoint molecules through RNA stability control, ubiquitination, or other post-transcriptional processes-remain to be fully elucidated and require targeted mechanistic studies using approaches such as RNA immunoprecipitation, RNA stability, and ubiquitination assays. In addition, despite validation in two glioma cell lines, *in vitro* models cannot fully recapitulate the cellular complexity and immune context of the glioma microenvironment. Finally, although the prognostic model was validated across multiple independent public datasets, prospective validation in well-characterized clinical cohorts will be essential to confirm its robustness and translational potential.

In summary, our findings demonstrate that MEX3 family genes are dysregulated in glioma and are closely linked to malignant progression, patient prognosis, and immune-related features. Among them, MEX3D emerges as a particularly promising prognostic marker with potential relevance to tumor-immune interactions. This study provides a comprehensive foundation for future mechanistic and translational investigations aimed at clarifying the role of MEX3 family members in glioma biology and exploring their potential as biomarkers or therapeutic targets.

## Data Availability

The datasets presented in this study can be found in online repositories. The names of the repository/repositories and accession number(s) can be found in the article/**Supplementary Material**.

## Supplementary material

Supplementary Figure 1 Associations between MEX3 family gene expression and clinicopathological and molecular characteristics of glioma. **(A)** Expression levels of MEX3 family genes across WHO tumor grades, showing grade-specific expression patterns. **(B)** Differential expression of MEX3 genes according to IDH mutation status. **(C)** Expression profiles of MEX3 family members among histological subtypes, including astrocytoma, oligodendroglioma, and glioblastoma. **(D)** Comparison of MEX3 gene expression between 1p/19q-codeleted and non-codeleted gliomas. **(E)** Expression patterns of MEX3 family genes across molecular subtypes (classical, mesenchymal, and proneural).

Supplementary Figure 2 External validation of MEX3 family gene upregulation in glioma. **(A)** Expression levels of MEX3A, MEX3B, MEX3C, and MEX3D in glioma and normal tissues in the CGGA cohort. **(B)** Validation of MEX3 family gene overexpression in glioma using the GSE16011 dataset.

Supplementary Figure 3 The ten most positively correlated and ten most negatively correlated genes for each MEX3 family member based on correlation analysis **(A-D)**.

Supplementary Figure 4 External validation of the prognostic risk model in independent CGGA cohorts. **(A)** Kaplan-Meier survival analysis showing a significant difference in overall survival between high- and low-risk groups in the CGGA_325 cohort. **(B)** Time-dependent ROC curves evaluating the predictive performance of the risk model for 1-, 3-, and 5-year overall survival in the CGGA_325 cohort. **(C)** Kaplan-Meier survival analysis demonstrating preserved risk stratification in the CGGA_693 cohort. **(D)** Time-dependent ROC curves assessing the prognostic accuracy of the risk model for 1-, 3-, and 5-year overall survival in the CGGA_693 cohort.
