# Supplementary figures and images for: Integrative bioinformatic and experimental analysis reveals prognostic and immunological roles of MEX3 family genes in glioma
Source: Front Immunol. 2026 Jan 14;16:1654036. doi: 10.3389/fimmu.2025.1654036 (PMC12847448; doi:10.3389/fimmu.2025.1654036)

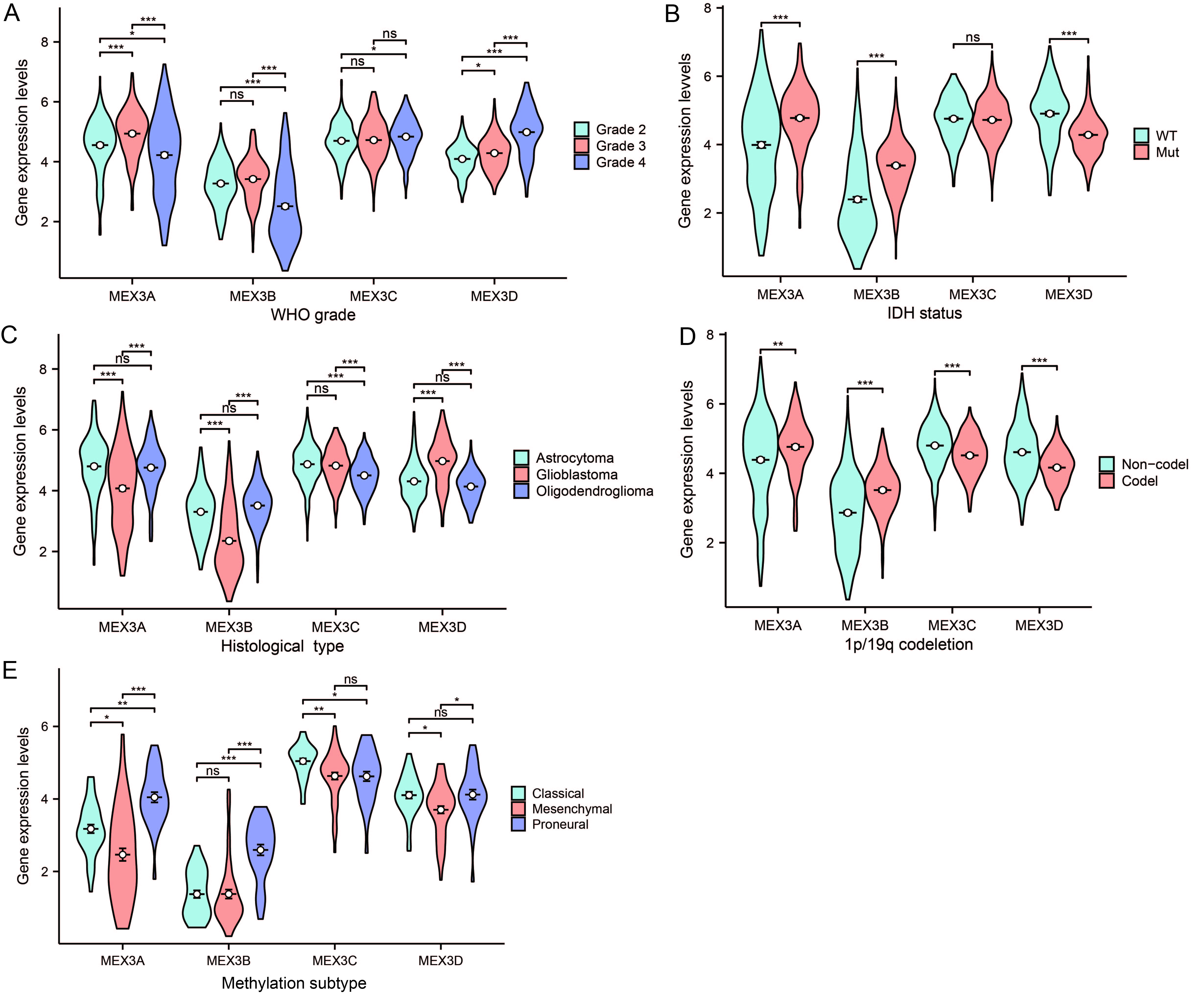

Supplement: Supplementary Figure 1 — Associations between MEX3 family gene expression and clinicopathological and molecular characteristics of glioma. (A) Expression levels of MEX3 family genes across WHO tumor grades, showing grade-specific expression patterns. (B) Differential expression of MEX3 genes according to IDH mutation status. (C) Expression profiles of MEX3 family members among histological subtypes, including astrocytoma, oligodendroglioma, and glioblastoma. (D) Comparison of MEX3 gene expression between 1p/19q-codeleted and non-codeleted gliomas. (E) Expression patterns of MEX3 family genes across molecular subtypes (classical, mesenchymal, and proneural). [file Image1.jpeg]

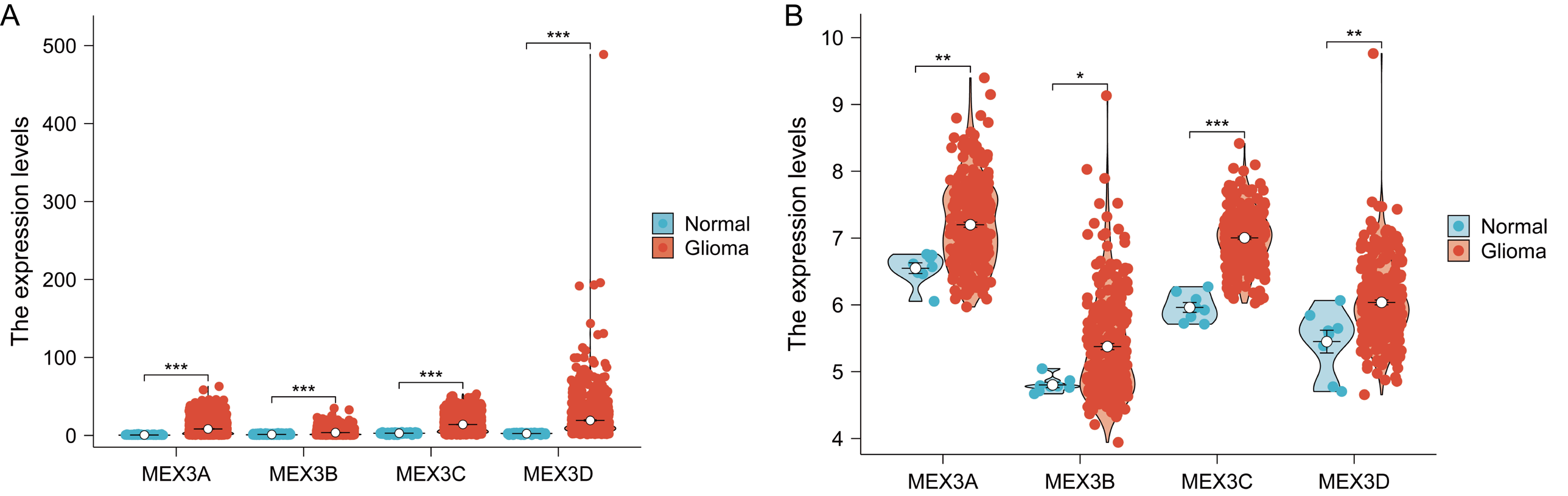

Supplement: Supplementary Figure 2 — External validation of MEX3 family gene upregulation in glioma. (A) Expression levels of MEX3A, MEX3B, MEX3C, and MEX3D in glioma and normal tissues in the CGGA cohort. (B) Validation of MEX3 family gene overexpression in glioma using the GSE16011 dataset. [file Image2.jpeg]

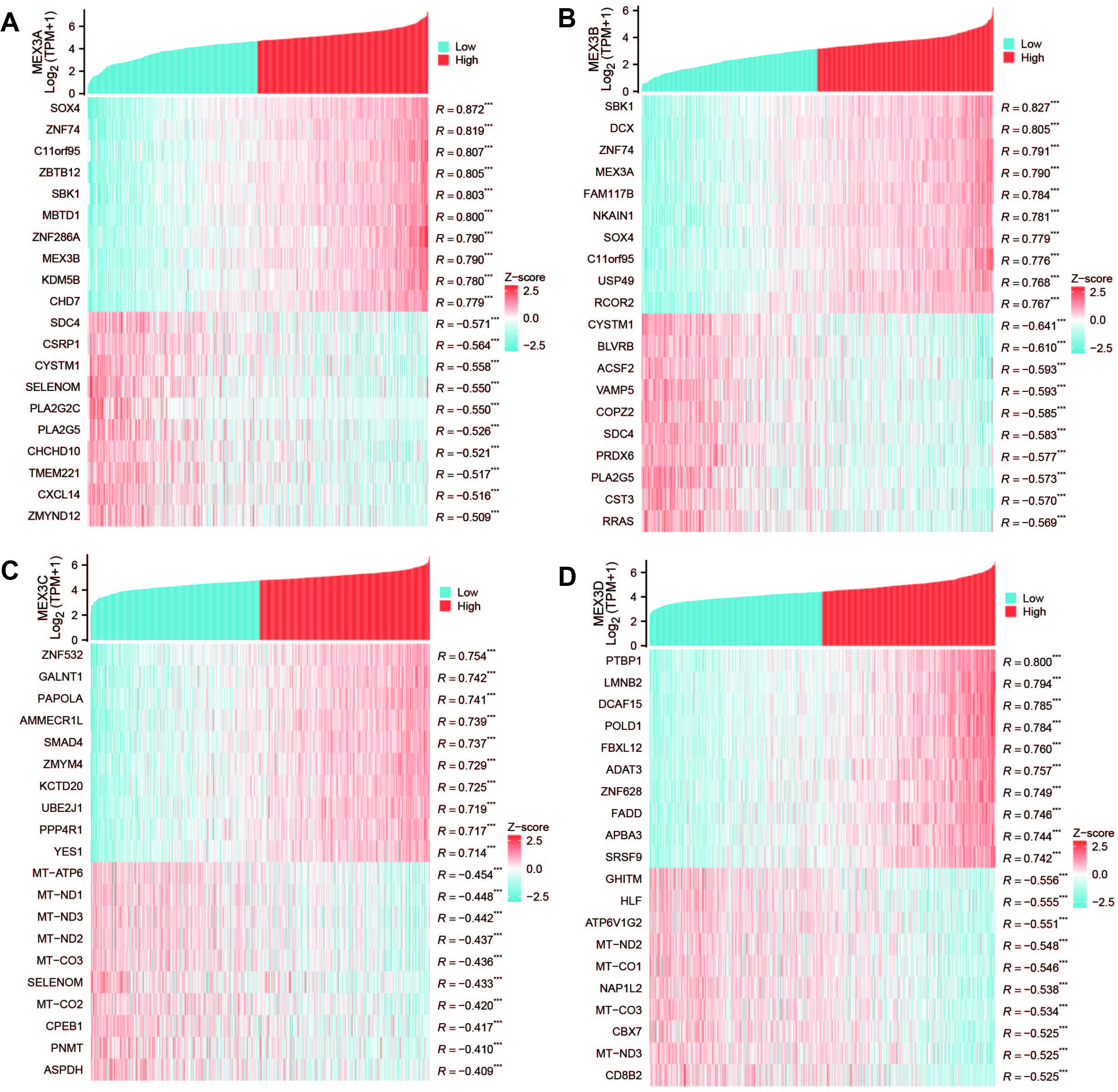

Supplement: Supplementary Figure 3 — The ten most positively correlated and ten most negatively correlated genes for each MEX3 family member based on correlation analysis (A-D). [file Image3.jpeg]
